# Targeting the blood–brain barrier with phytochemicals to attenuate vascular cognitive impairment: mechanisms and therapeutic potential across etiologies

**DOI:** 10.3389/fnut.2025.1696066

**Published:** 2026-01-23

**Authors:** Zixiang Jin, Xianglong Zhai, Yingfei Bai, Jun Hu, Haolu Yu, Jiajia Sang, Minghua Wu, Feng Hao

**Affiliations:** 1 Department of Integrated Traditional Chinese and Western Medicine, Peking University First Hospital, Beijing, China; 2 The First School of Clinical Medicine, Nanjing University of Chinese Medicine, Nanjing, China; 3 Jiangsu Provincial Hospital of Chinese Medicine, Nanjing, China; 4 College of Acupuncture-Moxibustion and Tuina, College of Health Preservation and Rehabilitation, Nanjing University of Chinese Medicine, Nanjing, China

**Keywords:** blood–brain barrier, Chinese herbal medicine, phytochemicals, vascular cognitive impairment, vascular dementia

## Abstract

Vascular cognitive impairment (VCI), a cognitive disorder arising from cerebrovascular pathology, currently lacks effective targeted therapies. Dysfunction of the blood–brain barrier (BBB) represents a pivotal event in VCI pathogenesis, characterized by tight junction degradation, neuroinflammation, and oxidative stress. Phytochemicals—including polyphenols, flavonoids, and polysaccharides—demonstrate promising multi-target potential in ameliorating VCI by preserving BBB integrity, mitigating neuroinflammation and oxidative stress, exerting neuroprotective effects, and modulating the gut–brain axis. However, the clinical translation of these compounds is currently impeded by low bioavailability and limited BBB permeability. Prioritizing the development of targeted delivery systems is essential to enhance the therapeutic efficacy and clinical utility of phytochemicals in the prevention and treatment of VCI.

## Graphical Abstract

**Figure:**
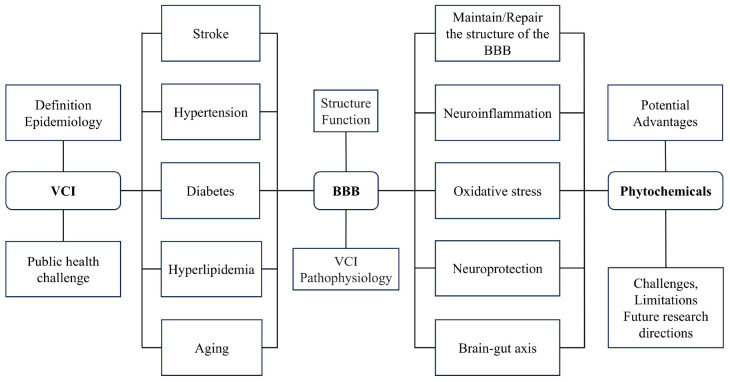
Targeting the blood-brain barrier with phytochemicals to attenuate vascular cognitive impairment.

## 1 Introduction

### 1.1 Vascular cognitive impairment and the public health burden

Vascular cognitive impairment (VCI) encompasses the full spectrum of cognitive dysfunction attributable to cerebrovascular disease (CVD) or the presence of vascular risk factors (1). A systematic review spanning 12 countries indicated that the prevalence of post-stroke VCI ranges from 20% to 80% (2). Furthermore, a multicenter cross-sectional study in China reported a VCI incidence of 78.7% among patients with a first-ever ischemic stroke, noting a significant association with a history of hypertension and diabetes (3). As VCI progresses, cognitive deficits may reach the threshold of dementia and significantly impair activities of daily living; this stage culminates in vascular dementia (VaD), the most severe phenotype and terminal stage within the vascular cognitive impairment spectrum (4).

VaD, recognized as the second most common form of dementia following Alzheimer’s disease (AD), exhibits varying prevalence across different regions. It accounts for approximately 15–20% of dementia cases in North America and Europe, rising to 30% in Asia and other developing countries (5). Current pharmacological interventions for VaD primarily rely on cholinesterase inhibitors and memantine—agents licensed for AD—yet their efficacy in treating vascular dementia remains limited. In contrast to the extensive study of AD, research elucidating the pathophysiology and potential pharmacotherapies for VaD remains comparatively scarce (6). Consequently, active mitigation of VCI risk factors and the implementation of targeted interventions during the early stages of impairment are essential to impede the progression to VaD.

### 1.2 Blood–brain barrier dysfunction: a key link in the onset and progression of vascular cognitive impairment

**FIGURE 1:**
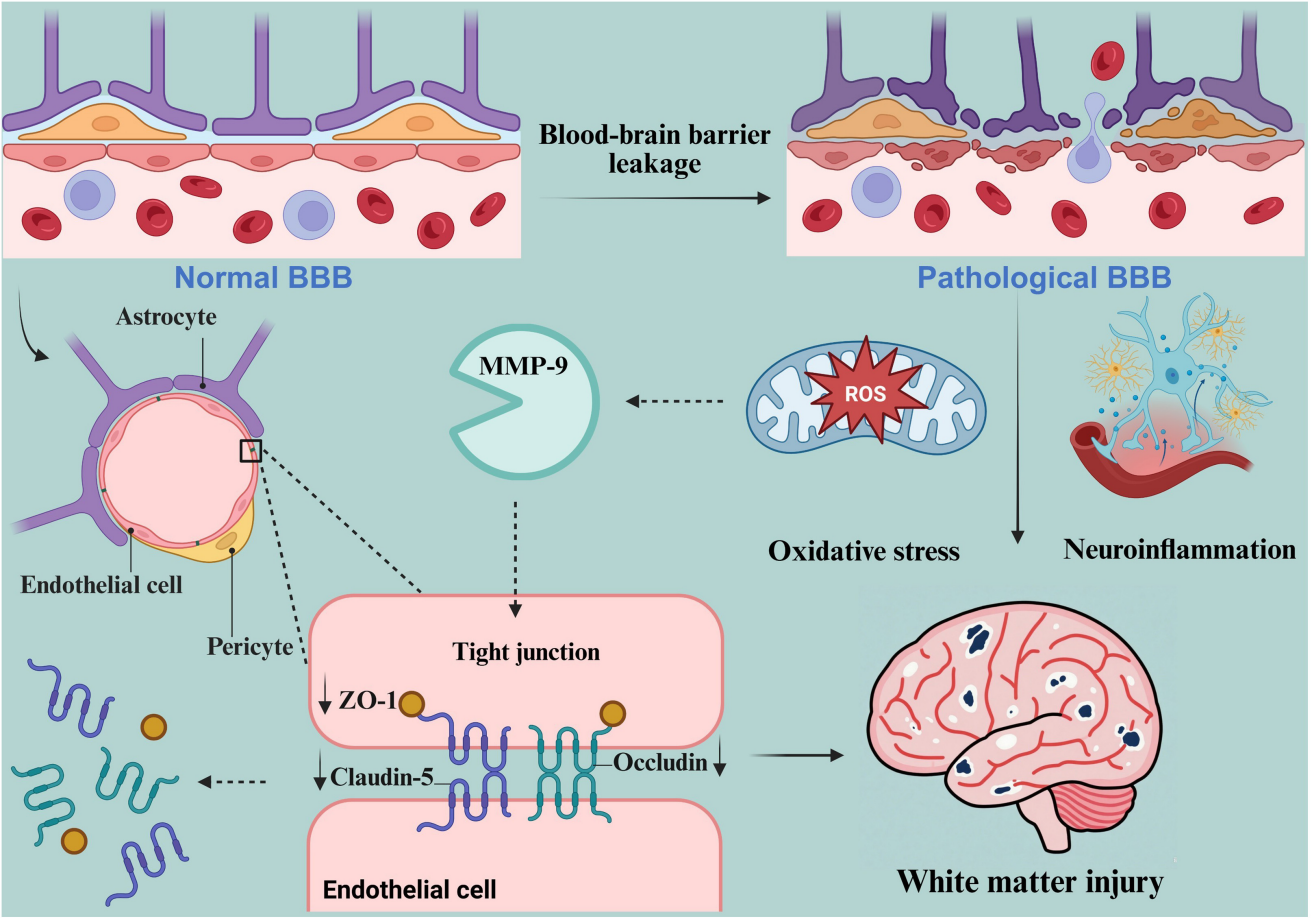
The mechanism of BBB dysfunction in VCI. Created in https://BioRender.com.

The blood–brain barrier (BBB) constitutes a selective interface between the plasma and brain parenchyma formed by the cerebral capillary endothelium and neuroglial cells, distinct from the barrier between the plasma and cerebrospinal fluid established by the choroid plexus. Composed of endothelial cells, tight junction (TJ) proteins, pericytes, astrocyte endfeet, and the basement membrane, the BBB acts as a gatekeeper between the systemic circulation and the central nervous system, selectively regulating the transit of substances (7). Brain capillary endothelial cells form the structural core of the BBB. In contrast to peripheral vascular endothelial cells, they are characterized by a lack of fenestrations, abundant mitochondria, and minimal vesicular transport activity. Furthermore, they establish a continuous intercellular barrier via TJs. This unique ultrastructure renders the BBB highly impermeable to most water-soluble substances, permitting only lipid-soluble small molecules to diffuse passively into the brain parenchyma (8). TJs are protein complexes comprising claudins, occludins, junctional adhesion molecules, and zonula occludens (ZO) proteins; their integrity directly dictates the permeability of the BBB (9). In patients with Mucopolysaccharidosis Type III A, occludin expression is significantly reduced in the striatum and hippocampus, while claudin-5 exhibits a downward trend across all brain regions. This region-specific expression pattern correlates closely with the clinical manifestations of cognitive impairment (10). Under conditions of chronic cerebral hypoperfusion (CCH), both the abundance and distribution of TJ proteins are altered. Specifically, the polar localization of ZO-1 in brain microvascular endothelial cells is disrupted, shifting from a concentrated distribution at membrane junctions to a diffuse cytoplasmic dispersion. This aberration in subcellular localization directly compromises the mechanical integrity of the BBB (11). Matrix Metalloproteinases (MMPs), a family of zinc-dependent endopeptidases, play a critical pathological role in VCI when their activity is dysregulated. Experimental models of ischemic stroke have demonstrated that MMP-9 directly impairs BBB structural integrity by degrading the TJ proteins claudin-5 and ZO-1 (12). Supporting the endothelium are pericytes, which occupy the space between endothelial cells and the basement membrane and extend projections covering approximately 30% of the capillary surface (13). They regulate cerebral hemodynamics by sensing and responding to blood flow fluctuations, modulating vasodilation and vasoconstriction to ensure stable perfusion (14). Furthermore, pericytes modulate neuroinflammation by regulating leukocyte infiltration (15). Astrocytic processes culminate in endfeet that firmly adhere to the capillary basement membrane. The basement membrane and its protein components provide a structural scaffold, binding the elements of the BBB together (16). The pathogenesis of VCI is inextricably linked to BBB impairment. Neuroinflammation and oxidative stress serve as the core pathological mechanisms driving this dysfunction, forming a complex positive feedback loop that synergistically exacerbates barrier collapse. This inflammatory cascade is initiated by the aberrant activation of microglia and astrocytes. In a rat model of chronic cerebral hypoperfusion (CCH) induced by bilateral carotid artery stenosis (BCAS), neuroinflammation manifested as glial hyperactivation and elevated levels of pro-inflammatory cytokines, specifically Tumor Necrosis Factor-alpha (TNF-α) and Interleukin-1 beta (IL-1β). These cytokines activate the nuclear factor kappa-B (NF-κB) pathway, precipitating the degradation of the tight junction (TJ) proteins claudin-5 and ZO-1, alongside the upregulation of matrix metalloproteinase expression. These alterations ultimately result in increased BBB permeability and cognitive dysfunction (17). Moreover, recent studies utilizing a BCAS model in *C57BL/6J* male mice confirmed that such excessive astrocytic activation promotes cytokine release, induces TJ degradation, and aggravates white matter injury, leading to cognitive decline (18). Concurrently, M1 microglial polarization amplifies this process by generating a “pro-inflammatory storm.” In a neonatal Sprague-Dawley rat model of traumatic brain injury, M1 polarization was significantly enhanced, resulting in the abundant production of TNF-α and IL-1β. Mechanistic investigations suggested that Deltex E3 ubiquitin ligase 1 directly drives microglial transformation toward the M1 phenotype by activating the NF-κB/Interferon Regulatory Factor 5 signaling pathway, thereby creating an inflammatory amplification cascade (19). Dysregulated iron metabolism represents a critical pathological link connecting oxidative stress to BBB injury. Following intracerebral hemorrhage, hemoglobin released from lysed erythrocytes is catabolized to heme, which is subsequently metabolized by Heme Oxygenase-1 to release free iron. This redox-active iron potently catalyzes the generation of hydroxyl radicals via the Fenton reaction (20). The overproduction of reactive oxygen species (ROS) in BBB endothelial cells initiates a deleterious cascade: it causes oxidative injury to cellular components, degrades TJ proteins, induces endothelial apoptosis, and activates MMPs, collectively culminating in BBB disruption (21, 22). Neurovascular unit dysfunction, precipitated by elevated BBB permeability, ultimately impairs cognitive function via white matter injury—a critical pathological mechanism driving VCI progression. Both neuroimaging and clinical investigations have corroborated that white matter injury represents a core downstream manifestation of BBB compromise. In a large community-based cohort of middle-aged adults, White Matter Hyperintensities (WMH) were predictive of an increased risk of stroke, amnestic mild cognitive impairment, dementia, and mortality (23). Dynamic contrast-enhanced MRI revealed that the most pronounced elevation in BBB permeability within WMH lesions in VCI patients occurs at the lesion core. This spatial distribution underscores the intimate pathological association between BBB dysfunction and the genesis of white matter lesions (24). In summary, the core mechanisms of BBB dysfunction in VCI—encompassing tight junction degradation, MMP activation, and the vicious cycle of neuroinflammation and oxidative stress—are collectively illustrated in Figure 1. The white matter damage elicited by BBB disruption encompasses complex molecular and cellular mechanisms. Hypertension serves as a prototypical precipitant: under hypertensive conditions, BBB dysfunction facilitates perivascular collagen deposition; this is compounded by chronic hypoperfusion, which further exacerbates permeability and ultimately induces structural damage to white matter (25). Additionally, under CCH conditions, TNF-α activation induces the downregulation of the TJ protein claudin-5, an alteration strongly correlated with the formation of deep brain white matter lesions (26). During the progression of white matter injury, Oligodendrocyte Precursor Cells (OPCs) contribute to the early modulation of BBB opening via the secretion of MMP-9. Animal studies have confirmed that OPCs specifically upregulate MMP-9 expression during the acute injury phase, resulting in the degradation of the TJ protein occludin and subsequent neutrophil infiltration. This process compromises BBB architecture, perpetuates a vicious cycle, and exacerbates vascular pathology (27). The long-term sequelae of white matter injury are characterized by the disruption of neural functional networks. Myelin loss and reduced axonal density impede neural signal transmission and impair sensorimotor integration, deficits that underpin the executive dysfunction and reduced information processing speed frequently observed in VCI patients (28). Autopsy studies corroborate this perspective, revealing widespread perivascular space enlargement and myelin debris deposition in the brains of VaD patients exhibiting white matter lesions. These pathological alterations were significantly correlated with ante-mortem cognitive performance (29).

In pathological conditions such as Chronic Cerebral Hypoperfusion (CCH), the expression levels of major TJ proteins—specifically occludin, claudin-5, and ZO-1—are downregulated, while MMP activity becomes dysregulated. This degradation of TJ architecture directly compromises the physical barrier function of the BBB. Subsequently, neuroinflammation and oxidative stress establish a positive feedback loop, synergistically amplifying cellular injury and markedly increasing BBB permeability. Ultimately, BBB breakdown disrupts neurovascular unit homeostasis. Driven by the cumulative effects of hypertension-induced perivascular collagen deposition, persistent injury from chronic hypoperfusion, and OPC-mediated MMP-9 secretion, white matter damage progressively intensifies. The resulting myelin loss and reduced axonal density impede neural signal transmission, precipitating cognitive decline and driving the onset and progression of VCI.

### 1.3 Potential and advantages of phytochemicals as a strategy for preventing and treating VCI

Phytochemicals are bioactive compounds derived from plants that exert beneficial physiological effects on living organisms. Constituting the essential pharmacological foundation for the therapeutic efficacy of numerous Traditional Chinese Medicines (TCM), these compounds regulate multiple physiological functions at relatively low doses. This diverse class encompasses molecules such as bioactive peptides, glycosides, polyphenols, volatile oils, and other secondary metabolites (30).

Owing to their natural origin, structural diversity, and broad pharmacological activities, phytochemicals represent a promising strategy for managing VCI. In the context of dietary intake, studies have consistently demonstrated that the consumption of fruits and vegetables—the most common sources of plant-based nutrients—is closely associated with a reduced risk of cognitive impairment (31–33). As an initial insight, a cross-sectional study of individuals aged 55 years or older demonstrated that the prevalence of Mild Cognitive Impairment (MCI) was significantly lower among those who consumed green vegetables daily compared to those who did not (OR = 0.218, 95% CI: 0.116–0.411, *p* < 0.001). This relationship remained significant after adjusting for age, education level, and other confounding factors, suggesting that daily vegetable consumption is a feasible prophylactic strategy against cognitive decline in older adults (34). Corroborating this association via a prospective design, a second cohort study involving 16,737 participants revealed that the quantity and variety of fruit and vegetable consumption in midlife were inversely correlated with the risk of late-life cognitive impairment. The odds ratios (OR) for the highest versus the lowest quartiles of intake were 0.83 (95% CI: 0.73–0.95, *p* = 0.006) for fruits and 0.76 (95% CI: 0.67–0.87, *p* < 0.001) for vegetables. Furthermore, dose-response analyses indicated that each additional daily serving of fruit or vegetables reduced the odds of cognitive impairment by 22% and 15%, respectively, with high consumption of both groups yielding a 23% risk reduction (35). This protective relationship is further substantiated at the meta-analytic level. A systematic review of six studies (*n* = 17,537) utilizing a random-effects model demonstrated that fruit and vegetable intake was significantly inversely associated with cognitive impairment (OR = 0.79, 95% CI: 0.67–0.93, *p* = 0.006). Subgroup analysis specifically identified this significant association within Chinese populations (OR = 0.74, 95% CI: 0.61–0.89, *p* = 0.002) (36). Critically, this benefit extends to the “oldest-old” population. In a longitudinal study of 4,749 cognitively normal adults aged 80 years and older, habitual consumers of fruits, vegetables, meat, and soy-derived products experienced 21, 25, 17, and 20% lower risks of developing cognitive impairment, respectively, compared to infrequent consumers (37). Finally, the most comprehensive synthesis of evidence to date—a meta-analysis of 16 studies comprising 64,348 individuals and 9,879 cases—identified a linear trend wherein increased fruit and vegetable intake correlated with a lower prevalence of cognitive impairment (OR = 0.79, 95% CI: 0.76–0.83). When analyzed separately, both fruit (OR = 0.83, 95% CI: 0.77–0.89) and vegetable (OR = 0.75, 95% CI: 0.70–0.80) consumption significantly decreased risk. This association was evident for cognitive impairment and dementia but not for Alzheimer’s disease specifically, providing robust support for the protective effect of phytochemical-rich diets against VCI (38).

In addition to vegetables and fruits, other plant-based dietary components hold significant promise for combating VCI. For instance, the National Institute for Longevity Sciences-Longitudinal Study of Aging, a Japanese study involving subjects aged 60–81 years, found that in women, a one-standard-deviation increase in the intake of total beans, total soybeans, and total soy isoflavones was associated with risk reductions of 52, 49, and 45%, respectively. These findings suggest that soy and its constituent isoflavones may exert a protective effect on cognition in older women (39). Beyond legumes, lipid-rich plant foods have also demonstrated therapeutic potential. Research focusing on patients with MCI has highlighted the efficacy of olive oil. Results indicated that daily consumption of extra virgin olive oil or refined olive oil for six months improved Clinical Dementia Rating and behavioral scores. Notably, extra virgin olive oil significantly reduced BBB permeability and enhanced brain functional connectivity, benefits potentially attributable to its phenolic content (40). Collectively, these results underscore the potential of phytochemicals in mitigating VCI risk. The advantage of this approach lies not only in the accessibility of these foods but also in their pleiotropic mechanisms of action, particularly regarding BBB targeting. Consequently, this represents a natural, food-based strategic paradigm for the prevention and intervention of VCI.

## 2 Targeting the BBB for the prevention and treatment of VCI: strategies and evidence across different etiological backgrounds

### 2.1 Stroke

Stroke, a major neurological disorder characterized by high global morbidity and disability rates, serves not only as the second leading cause of death following infectious diseases and the fourth leading cause of disability (41) but also as a critical risk factor for the development of VCI (42). Extensive research has demonstrated that neuroinflammation, blood pressure fluctuations, and hyperglycemia are associated with poor prognosis in patients with acute ischemic stroke (43–45). Regarding cognitive sequelae, clinical studies indicate that 20–80% of ischemic stroke survivors experience immediate or delayed VCI (46). This association is further substantiated by community-based cohort studies reporting that 42.3% of individuals without significant neurodegenerative pathology developed VCI, a progression primarily driven by severe cerebrovascular pathology (47). In recent years, the mechanistic understanding of this relationship has deepened, with increasing evidence identifying BBB disruption as a pivotal process linking stroke to VCI (48). From a neuroimaging perspective, infarct volume and extent, the severity of white matter lesions, and the progression of cerebral atrophy represent critical features reflecting the pathological link between post-stroke BBB disruption and VCI (49). Patients with post-stroke VCI exhibit not only larger volumes of frontal WMH but also significantly exacerbated astrocytic degeneration, accompanied by the disruption of neuroglial-vascular interactions and BBB damage (50). The clinical relevance of early barrier compromise is highlighted by findings that BBB disruption detected via MRI within the first 3 h of symptom onset in acute ischemic stroke patients is associated with the occurrence of vasogenic edema (51). Mechanistically, inflammation-driven BBB injury in ischemic stroke involves oxidative stress, elevated production of MMPs, and microglial activation (52). Central to this process is the disruption of TJs, which constitutes a primary cause of increased paracellular BBB permeability post-stroke. During this process, TJ proteins undergo a cascade of progressive alterations, including protein modification, translocation, and degradation (53). A large-scale prospective cohort study from the UK Biobank identified a non-linear association between coffee and tea consumption and the risk of stroke and dementia. The lowest Hazard Ratios (HRs) for incident disease were associated with a combined daily intake of approximately 4–6 cups of coffee and tea. Furthermore, this combined consumption pattern was associated with a decreased risk of ischemic stroke and VaD; notably, the lowest risk for post-stroke dementia was observed at a total daily intake of 3–6 cups (HR 0.52, 95% CI 0.32–0.83; *p* = 0.007) (54). Another clinical trial involving patients with post-stroke VCI highlighted the cognitive benefits of Gotu Kola Extract. After a 6-week intervention, the improvement observed in the 1,000 mg/day Gotu Kola Extract group was significantly superior to that of the folic acid group across multiple cognitive domains (55).

### 2.2 Hypertension

Hypertension constitutes the most significant risk factor for cerebral small vessel disease (SVD). The systolic hypertension in Europe (Syst-Eur) dementia trial randomized 2,418 non-demented participants with isolated systolic hypertension to antihypertensive treatment or control groups. After a median follow-up of 2 years, the incidence of dementia was significantly reduced by 50% in the active treatment group (56). This finding establishes hypertension as a key modifiable risk factor for VaD and underscores its underlying vascular pathological basis: hypertension disrupts BBB integrity by inflicting sustained damage on cerebral small vessels, thereby inducing white matter injury and driving VCI (57). This vascular pathology extends beyond the microcirculation to involve the macrovasculature. Long-term midlife hypertension promotes atherosclerosis, which, when compounded by cerebral hypoperfusion secondary to late-life hypotension, is directly associated with VCI and VaD (58). A distinct dose-response relationship exists between systemic atherosclerotic burden and VCI risk. Compared to individuals without atherosclerosis, the risk of cognitive impairment was nearly tripled in those with multi-vessel disease, increasing by 76% for each additional affected vascular bed (59). BBB disruption represents a critical stage in the progression of hypertension-related SVD, mediated by multifaceted mechanisms. First, mechanical shear stress disrupts the membrane localization of endothelial TJ proteins, leading to paracellular leakage (60). Second, hypertension-induced oxidative stress—characterized by the overproduction of ROS—activates endothelial apoptotic pathways and depletes antioxidant systems, further compromising the endothelial barrier (61). Furthermore, chronic hypertension stimulates microglial activation, resulting in the production of pro-inflammatory cytokines and MMPs (62). MMPs can directly degrade TJ proteins and the basement membrane, thereby exacerbating BBB leakage (63). Specifically, MMP-3 and MMP-9 cleave the extracellular domain of claudin-5; this action not only disrupts intercellular junctions but also induces the degradation of basement membrane components, including laminin and collagen IV, further damaging the BBB (64). Elevated BBB permeability precipitates vasogenic edema and facilitates the influx of circulating inflammatory mediators and neurotoxic substances into the brain parenchyma, thereby triggering local inflammatory cascades (65). As small vessel remodeling and BBB disruption progress, they induce white matter injury—manifesting primarily as WMH—which directly contributes to VCI. Supporting this pathway, a clinical study of 155 participants linked cardiovascular risk factors to increased WMH volume. Furthermore, mechanistic evidence from human brain microvascular endothelial cells (hBMEC) demonstrated that these risk factors impair BBB integrity, as evidenced by decreased transendothelial electrical resistance (TEER), thereby contributing to WMH and VCI. Thus, early management of hypertension is essential (66, 67). Phytochemicals have been confirmed to ameliorate these conditions. For instance, *Myrtus communis* extract demonstrated cognitive protective effects comparable to ramipril in a renovascular hypertension model. It reduces angiotensin II levels by inhibiting angiotensin-converting enzyme activity, thereby mitigating inflammation, oxidative stress, and MMP-13 expression while upregulating the anti-inflammatory cytokine IL-10 (68).

### 2.3 Diabetes mellitus

Diabetes mellitus, particularly type 2 diabetes, is recognized as a potent risk factor for VCI (69). A cardinal pathophysiological mechanism involves the compromise of BBB integrity, leading to cerebral blood flow disturbances that precipitate neuronal damage. Hyperglycemia constitutes the hallmark of diabetes. Under these conditions, glucose reacts with proteins to form AGEs, which accumulate in cerebral microvascular endothelial cells. Binding of AGEs to their receptors activates the NF-κB pathway, triggering oxidative stress, inflammation, and cytoskeletal contraction; this degrades TJ proteins and enhances barrier permeability (70). Beyond AGE formation, hyperglycemia aberrantly activates the polyol pathway, metabolizing glucose into sorbitol. Intracellular sorbitol accumulation elevates osmotic pressure, causing endothelial swelling and functional loss. Concurrently, this pathway consumes NADPH, exacerbating oxidative stress. Recent findings indicate that high glucose levels drive this pathway to cause cellular injury, an effect attenuated dose-dependently by aldose reductase inhibitors (71). Hyperglycemia also modulates the generation of ROS and Reactive Nitrogen Species (RNS), leading specifically to endothelial cell destruction, numerical reduction, and impaired physiological Nitric Oxide (NO) production. Since NO mediates vasodilation, its diminution compromises vascular relaxation and BBB structural integrity (72). Furthermore, hyperglycemia impairs endothelial mitochondrial function (73) and promotes the release of vasoconstrictors such as Endothelin-1 (ET-1). This leads to aberrant cerebrovascular contractility, hypoperfusion, and secondary endothelial damage driven by ischemia and hypoxia. ET-1 is a potent vasoconstrictive peptide, and its plasma levels are positively correlated with microangiopathy in patients with type 2 diabetes. Beyond its direct vasoconstrictive effects, elevated ET-1 may further impair endothelial function by inhibiting NO production (74). Studies utilizing diabetic endothelial cells have observed increased expression of mitochondrial fission proteins (Fis1, Drp1). This upregulation leads to mitochondrial fragmentation and elevated ROS generation, impairing eNOS activation. Notably, silencing Fis1 or Drp1 reverses these alterations, suggesting that aberrant mitochondrial fission represents a pivotal mechanism in diabetic endothelial dysfunction (75). In contrast to these deleterious effects, insulin exerts a protective influence. Insulin promotes endothelial repair and attenuates BBB injury by activating the PI3K/Akt pathway. In rat models, insulin attenuated burn serum-induced reductions in ZO-1 expression and increased TEER, effects abrogated by PI3K inhibitors (76). Mechanistically, insulin activates Akt and phosphorylates eNOS at Ser1177, doubling NO production. Furthermore, it enhances endothelial barrier function by modulating the phosphorylation status of the TJ protein ZO-1; crucially, this effect is completely abolished in Akt1 knockout mice (77). However, central insulin resistance compromises these beneficial actions. Finally, hyperglycemia reprograms astrocytes, inducing abnormal activation and disrupting end-foot architecture. Astrocyte-derived inflammatory mediators further aggravate neuronal injury (78, 79). In the *5xFAD* mouse model, reduced secretion of Brain-Derived Neurotrophic Factor (BDNF) from astrocytes results in a 30% decrease in neuronal dendritic spine density and impaired cognitive function. Hyperglycemia exacerbates this pathological cascade by inhibiting astrocytic BDNF synthesis (80).

### 2.4 Hyperlipidemia and obesity

Hyperlipidemia constitutes a pivotal risk factor for VCI. Epidemiological evidence from a cohort study involving 7,087 community-dwelling adults aged over 65 years utilized Cox proportional hazards models to demonstrate that metabolic syndrome increased the risk of VaD within four years; notably, elevated triglyceride levels were significantly associated with VaD risk (HR = 2.27, 95% CI 1.16–4.42, *p* = 0.02) (81). At the molecular level, elevated circulating free fatty acids and aberrant cerebral lipid accumulation directly induce cerebrovascular endothelial dysfunction, ultimately compromising BBB integrity (82, 83). Consistently, obese animal models exhibit downregulated expression of TJ proteins (ZO-1, occludins) and Glucose Transporter 1 (GLUT1), alongside upregulated aquaporin-4; collectively, these alterations contribute to structural and functional BBB impairment (84). This compromised barrier milieu is further exacerbated by high-fat diet-driven chronic inflammation, which sustains microglial activation, promotes the release of pro-inflammatory mediators, and diminishes BDNF levels, thereby intensifying neuroinflammation and oxidative stress to ultimately precipitate VCI (85, 86). The detrimental impact of hyperlipidemia extends beyond direct cerebrovascular effects, acting as a driver of polyvascular pathology. In *ZSF1* obese rat models, aberrant cerebral blood flow combined with endothelial dysfunction leads to elevated BBB permeability and hyperperfusion—changes directly linked to cognitive impairment.

### 2.5 Aging

Aging represents a primary etiological factor in VCI. Endothelial cells undergo senescence, a mechanism identified as a significant driver of BBB disruption and cognitive decline (87). Specifically, aging results in diminished endothelial proliferation and increased apoptosis, precipitating the widening of intercellular gaps (88). Concurrently, aging downregulates endothelial apolipoprotein E (ApoE) expression (89). This deficiency triggers the excessive release of cyclophilin A (CypA), which perturbs TJ protein localization and activates MMPs to degrade the basement membrane (90). Furthermore, aging compromises key transporter systems, including the glucose transporter GLUT1 and the efflux pump P-glycoprotein (P-gp). In aged mice, GLUT1 downregulation results in diminished glucose uptake and cerebral ATP levels (91). Similarly, P-gp function declines with age; canine models show a 72% reduction in P-gp expression in very old dogs, compromising metabolic waste clearance (92). Data from male Wistar rats further confirm that cerebral P-gp expression decreases with aging, potentially facilitating paracellular transport and increasing BBB permeability (93). In light of these mechanisms, plant-derived bioactive components demonstrate significant potential. A meta-analysis of 27 RCTs (*n* = 1,961) demonstrated that adjuvant therapy with Chinese herbal medicine significantly improved clinical efficacy in senile vascular dementia compared to conventional pharmacotherapy alone (OR = 2.98) (94). Beyond formulations, specific phytochemicals exhibit efficacy; for instance, cocoa flavanols enhance cerebral blood flow via NO-dependent vasodilation (95). Regarding cognitive impairment in senescence-accelerated mouse prone 8 (SAMP8) mice, *Dendrobium officinale* polysaccharide inhibits hippocampal microglial activation and downregulates pro-inflammatory factors, thereby mitigating inflammatory BBB injury (96). Similarly, in D-galactose-induced aging rats, *Astragalus* polysaccharide modulates the PI3K/Akt and NAMPT/SIRT1 pathways, regulates the TERT/p53 axis, alleviates neuronal degeneration in hippocampal subregions, and reduces cerebral oxidative stress, thereby establishing a microenvironment conducive to BBB maintenance and cognitive enhancement (97).

## 3 Key mechanisms of phytochemicals targeting the blood–brain barrier for improving vascular cognitive impairment

**FIGURE 2:**
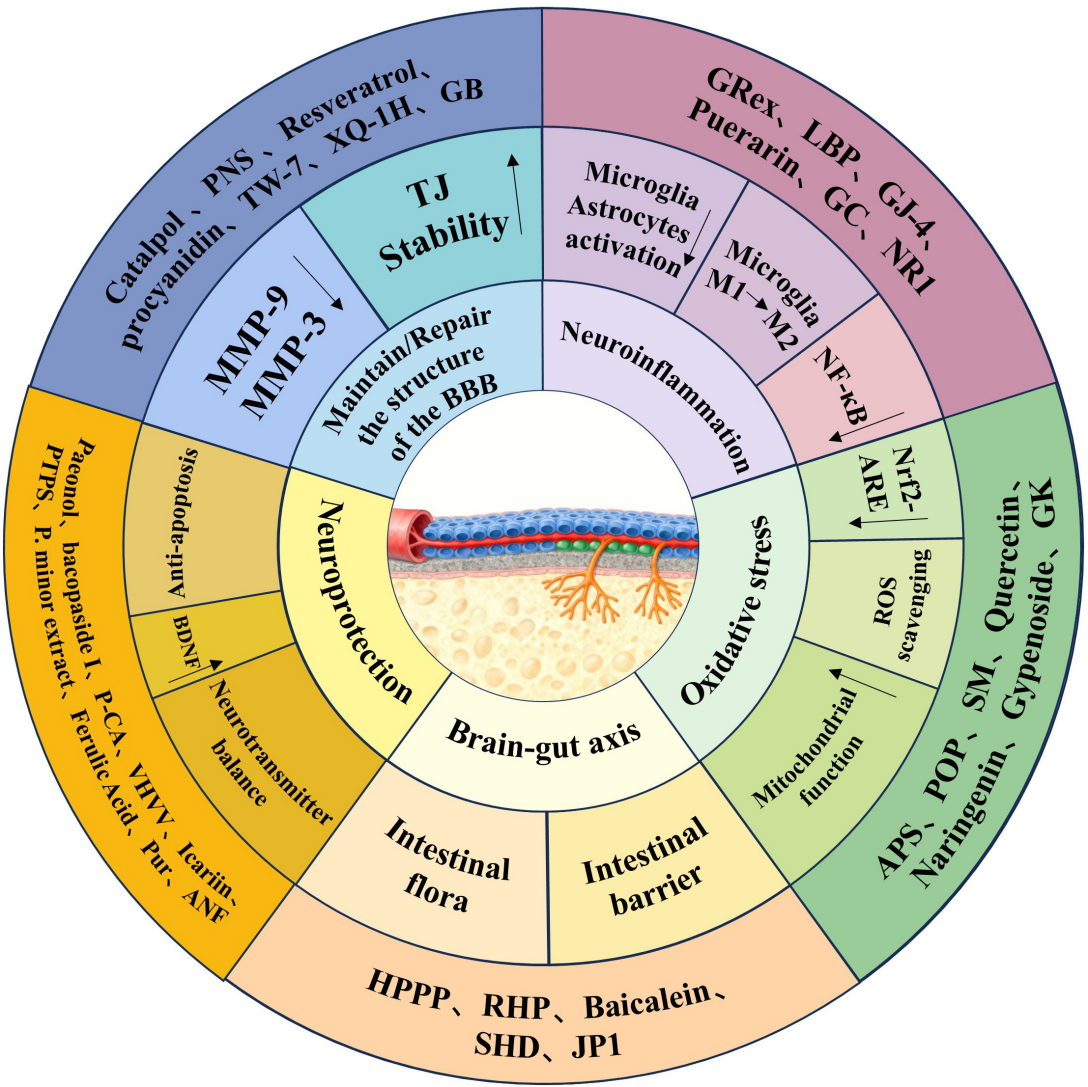
The mechanism of phytochemicals in improving VCI by Targeting the BBB.

Although extensive research has demonstrated the considerable potential of plant-derived nutrients in alleviating VCI, the strength and consistency of the evidence vary. Validating the efficacy of these compounds requires rigorous future investigation. However, emerging research increasingly points to a shared therapeutic mechanism: the targeting of the BBB as a core protective locus. As summarized in Figure 2, phytochemicals can protect the BBB and ameliorate VCI through multiple, synergistic mechanisms, including enhancing tight junction stability, inhibiting matrix metalloproteinases, mitigating neuroinflammation and oxidative stress, exerting neuroprotective effects, and modulating the gut-brain axis. The following sections elaborate on these key mechanisms.

### 3.1 Restoration of the structural and functional integrity of the BBB

Structural and functional compromise of the BBB constitutes a central pathological mechanism underlying VCI, spanning the pathogenesis and progression of diverse etiologies, including stroke, hypertension, diabetes mellitus, hyperlipidemia, and aging. Consequently, elucidating the specific mechanisms by which phytochemicals target the BBB to ameliorate VCI represents a pivotal step in advancing prevention and treatment strategies. First, phytochemicals directly stabilize the physical barrier of the BBB by reinforcing TJs and the cytoskeleton. A critical regulatory pathway is RhoA/ROCK; its overactivation precipitates TJ dissociation. Catalpol, from *Rehmannia glutinosa*, counteracts this by downregulating RhoA/ROCK2 expression and upregulating key TJ proteins (98). Similarly, *Panax notoginseng* saponins (PNS) reverse the downregulation of ZO-1 and claudin-5 during ischemia-reperfusion injury, thereby preserving TJ integrity (99). Beyond modulating protein expression, phytochemicals also maintain the conformational stability of TJ proteins. For instance, resveratrol prevents the high-fat diet-induced depolymerization of occludin and ZO-1, inhibiting their transition from a continuous linear arrangement to a discontinuous punctate distribution; this stabilizes their polymerization state and barrier function without altering total protein levels (100). Second, phytochemicals preserve BBB structural integrity by inhibiting MMPs. A study utilizing a model of Chemotherapy-Induced Cognitive Impairment (CICI) demonstrated that the chemotherapeutic agent cisplatin induces an upregulation of MMP-9 activity, thereby disrupting the BBB and precipitating cognitive deficits. Grape seed procyanidins have been shown to ameliorate cognitive impairment, restore BBB integrity, and efficiently inhibit the enzymatic activity of MMP-9 (101). Although not specific to VCI, the mechanistic insights derived from these models offer valuable guidance for therapeutic development. For instance, the walnut-derived peptide TWLPLPR demonstrates potential in inhibiting the NF-κB/MMP-9 signaling pathway; however, current evidence is primarily derived from Aβ25-35-injured bEnd.3 cells, a model that does not fully recapitulate the complex pathology of VaD. Consequently, direct *in vivo* evidence for TWLPLPR remains to be established (102). This inhibitory mechanism has also been validated using 10-O-(N, N-dimethylaminoethyl)-ginkgolide B methanesulfonate (XQ-1H), a derivative of Ginkgolide B. In an animal model of hyperlipidemia combined with ischemic stroke, XQ-1H inhibits MMP-9 overexpression, thereby preserving endothelial ultrastructure, reducing cerebral edema, and maintaining the physical barrier function of the BBB (103). Distinct from MMP inhibition, certain phytochemicals promote angiogenesis to facilitate BBB reconstruction. In Oxygen-Glucose Deprivation/Reperfusion (OGD/R) and Middle Cerebral Artery Occlusion (MCAO) models, GB inhibits endothelial Creatine Kinase B (CKB), triggering signaling cascades that enhance endothelial proliferation, migration, and tube formation. This activity increases microvessel density and vascular surface area within ischemic regions, thereby restoring cerebral blood flow and establishing a structural foundation for cognitive recovery (104).

### 3.2 Mitigation of neuroinflammation

Phytochemicals preserve BBB integrity and ameliorate VCI by suppressing neuroinflammation via multifaceted mechanisms. including the inhibition of glial hyperactivation and the modulation of cytokine networks. The Methanolic Extract of *Glycyrrhizae Radix et Rhizoma* (GRex) has demonstrated significant neuroprotective efficacy in a mouse model of Middle Cerebral Artery Occlusion (MCAO). At a dosage of 125 mg/kg, GRex effectively reduced cerebral edema and infarct volume following ischemia-reperfusion, thereby ameliorating post-stroke VCI. The underlying mechanism is attributed to the downregulation of astrocytic and microglial activation (105). An illustrative study by Zhao et al. (106) demonstrated that *Lycium barbarum* polysaccharides enhance cortical blood flow and mitigate memory and motor coordination deficits in mice with focal cerebral ischemia. These therapeutic benefits were attributed to the dual inhibition of microglial and astrocytic hyperactivation, as well as the suppression of MCAO-induced P65 NF-κB and P38 MAPK signaling pathways; collectively, these actions prevented the upregulation of hippocampal pro-inflammatory mediators and exerted neuroprotective effects (106). Beyond suppressing activation, certain phytochemicals actively reprogram microglial phenotypes. For instance, the *Gardenia jasminoides* J. Ellis extract GJ-4 improved cognitive function by activating PPAR-γ, promoting a shift from the pro-inflammatory M1 to the anti-inflammatory M2 microglial phenotype (107). The NF-κB pathway and NLRP3 inflammasomes serve as critical regulatory nodes. Sustained NF-κB activation promotes the transcription of pro-inflammatory cytokines such as TNF-α and IL-6, which directly disrupt TJs in BBB endothelial cells (108), while NLRP3 inflammasome activation intensifies the inflammatory cascade (109). Phytochemicals can intercept inflammatory signaling by targeting these pathways. In a rat model of ischemia-reperfusion injury, Puerarin inhibits the TLR4-mediated MyD88-dependent signaling pathway, downregulates the transcriptional activity of NF-κB, and reduces the release of the pro-inflammatory cytokine TNF-α; this suppression of excessive post-ischemic inflammation alleviates neurological deficits, cerebral infarction, and cerebral edema (110). In a Middle Cerebral Artery Occlusion/Reperfusion (MCAO/R) rat model, Ginkgolide C significantly alleviated neurological deficits, reduced cerebral infarct volume, and mitigated brain edema by inhibiting the CD40/NF-κB signaling pathway and suppressing the associated release of pro-inflammatory cytokines such as TNF-α and IL-6 (111). Similarly, Notoginsenoside R1 (NR1) exerts anti-inflammatory and neuroprotective effects by inhibiting the canonical TLR4/MyD88/NF-κB inflammatory signaling pathway and reducing pro-inflammatory cytokine release, thereby significantly decreasing cerebral infarct size, alleviating cerebral edema, and improving neurological function (112).

### 3.3 Alleviation of oxidative stress

Oxidative stress and neuroinflammation constitute a vicious cycle that serves as a core mechanism driving BBB dysfunction. A diverse array of phytochemicals has demonstrated robust and reproducible antioxidant properties across multiple experimental models. These compounds ameliorate VCI by enhancing endogenous antioxidant systems—notably the Nrf2-ARE pathway—scavenging ROS. In the *APP/PS1* mouse model, *Astragalus* polysaccharide was demonstrated to upregulate nuclear Nrf2 expression, restore SOD and glutathione peroxidase activities, reduce malondialdehyde (MDA) levels, and attenuate oxidative stress-induced damage to BBB endothelial cells (113). Similarly, *Polygonatum* polysaccharide significantly ameliorated cognitive function in D-galactose-induced aging rats by activating Nrf2, upregulating its expression, and downregulating ferroptosis-associated proteins. Moreover, this therapeutic effect was abrogated by Nrf2 inhibition, confirming that it operates via the Nrf2-ferroptosis pathway (114). In a transient Middle Cerebral Artery Occlusion (tMCAO) model, researchers observed that *Salvia miltiorrhiza* reduced post-ischemic glial hyperplasia and upregulated the expression of IL-6, TNF-α, and phosphorylated STAT3. It also reduced the levels of 4-hydroxynonenal and MDA in the penumbra, thereby inhibiting ferroptosis, alleviating ischemic neuronal injury, mitigating synaptic and neuronal loss, and improving post-stroke VCI (115). Another critical mechanism of phytochemicals involves the direct scavenging of ROS and the inhibition of oxidative damage product formation. Quercetin is renowned for its free radical scavenging capacity and its ability to suppress microglial activation. Takizawa et al. (116) reported that, functioning as a potent free radical scavenger, quercetin specifically eliminates peroxynitrite, inhibits microglial activation and ROS production, and ameliorates ischemic white matter injury, thereby mitigating VaD. However, the specific efficacy of quercetin in established VaD models lacks consistent documentation, and mechanistic insights remain speculative, necessitating further investigation (116). Previous studies have indicated that Naringenin inhibits hippocampal oxidative stress by reducing ROS and MDA levels while enhancing antioxidant enzyme activity. It also suppresses the NF-κB signaling pathway by downregulating inflammatory mediators, thereby mitigating inflammatory responses. Furthermore, Naringin has been shown to upregulate NR1, NR2B, and synaptic proteins, promoting NMDA receptor signaling to improve VaD outcomes (117). Animal experiments have also confirmed that Gypenoside scavenges oxygen-free radicals, enhances antioxidant capacity, reduces lipid peroxidation and oxidative DNA damage, and inhibits astrocytic activation in the corpus callosum and optic tract, ultimately improving VCI (118). However, evidence for *Gynostemma pentaphyllum* remains limited, and its mechanism of action is not fully elucidated, which requires further in-depth exploration.

Furthermore, phytochemicals can mitigate ROS generation at the source by improving mitochondrial function. In both MCAO rat models and *in vitro* OGD/R neuron models, Ginkgolide K exerts neuroprotective effects against ischemic stroke by inhibiting GSK-3β-mediated mitochondrial hyper-fission and membrane permeability increases, thereby reducing cerebral infarct volume and neuronal apoptosis (119). Collectively, these findings indicate that phytochemicals support the prevention and treatment of VCI by activating antioxidant pathways such as Nrf2-ARE, directly scavenging ROS, improving mitochondrial function, and blocking oxidative stress-induced BBB damage through multiple mechanisms.

### 3.4 Neuroprotection

Dysfunction of the BBB compromises nutrient delivery to neural cells, subsequently precipitating neuronal apoptosis, synaptic impairment, and diminished neuroplasticity—processes that constitute critical mechanisms underlying cognitive decline in VCI. Phytochemicals facilitate the repair of neural function following BBB injury through direct neuroprotective effects, including the inhibition of apoptosis and the upregulation of neurotrophic factor expression, thereby ameliorating VCI. Anti-apoptotic activity represents a fundamental mechanism by which phytochemicals exert neuroprotection. In ischemic stroke models, neuronal apoptosis exacerbates injury to the neurovascular environment surrounding the BBB; phytochemicals can inhibit this process by modulating apoptosis-related proteins. In a rat model of tMCAO, Paeonol was confirmed to ameliorate memory impairment following subacute ischemic stroke by reducing the count of TUNEL-positive cells, downregulating mitochondrial Bax protein expression, and suppressing the cytoplasmic release of Apoptosis-Inducing Factor (120). The ethanolic extract of *Bacopa monnieri* effectively ameliorates cerebral ischemia-induced cognitive deficits and mitigates neuronal damage by activating the PKC and PI3K/Akt pathways. Furthermore, it reverses the Oxygen-Glucose Deprivation-induced reduction in the phosphorylation of the anti-apoptotic factor Akt (p-Akt), thereby upregulating p-Akt to enhance cell survival signaling (121). Beyond averting cell death, phytochemicals actively promote neuroplasticity by enhancing neurotrophic factor expression. BDNF is a pivotal regulator of neurogenesis and synaptogenesis; decreased expression is causally linked to cognitive dysfunction. Phytochemicals modulate BDNF to improve VCI. In a rat model of ischemic stroke, *P*-Coumaric Acid promotes hippocampal neurogenesis and improves spatial cognitive function by activating the BDNF/TrkB/AKT signaling pathway (122). The bioactive peptide VHVV, derived from soybean protein hydrolysate, possesses the capacity to cross the BBB. It ameliorates hypertension-mediated VCI and neuronal degeneration by upregulating BDNF expression, activating the cAMP Response Element-Binding protein (CREB) and PI3K-AKT-mTOR pathways, thereby promoting neuronal survival and reducing apoptosis (123).

Additionally, phytochemicals improve VCI by regulating neurotransmitter balance and enhancing neuroplasticity via multiple targets. Regarding the regulation of the core cholinergic system, various components have demonstrated positive effects; for instance, Icaritin enhances CREB phosphorylation to restore histone acetylation homeostasis within cholinergic circuits (124). Polysaccharides derived from *Polygala tenuifolia* maintain the balance between ACh and AChE, a mechanism linked to the upregulation of the BDNF/TrkB/ERK/CREB signaling pathway (125). Second, in maintaining the excitation/inhibition balance, distinct phytochemicals act on specific targets: the extract of *Persicaria minor* enhances hippocampal ACh and GABA levels and increases the expression of α5-GABA_A receptors to coordinate neural activity (126). High oleic peanuts primarily regulate the glutamate-to-GABA ratio to restore dynamic network balance (127). Puerarin directly inhibits the excessive efflux of excitatory amino acids induced by cerebral ischemia, thereby reducing excitotoxic injury (128). In addition to direct neurotransmitter regulation, certain phytochemicals afford indirect neuronal protection through antioxidant and anti-inflammatory mechanisms. For instance, alpha-naphtho flavone, found in Russian knapweed, not only improves cholinergic transmission but also alleviates oxidative stress and inflammation by increasing GSH levels and reducing lipid peroxidation (129).

### 3.5 Modulation of the brain-gut axis

Recent investigations have revealed that phytochemicals, in addition to directly targeting the BBB, inhibiting neuroinflammation, and attenuating oxidative stress to improve VCI, can also indirectly protect the BBB by regulating the Microbiota-Gut-Brain Axis (MGBA) and systematically remodeling the central nervous system microenvironment. The MGBA constitutes a critical pathway for maintaining bidirectional communication between the peripheral and central nervous systems. The gut microbiota modulates central nervous system (CNS) function via vagus nerve signaling, immune mediator release, and the secretion of microbial metabolites (130). Specifically, phytochemicals can mitigate persistent inflammatory insults to the BBB by repairing and reinforcing the physical barrier of the gut, thereby preventing the translocation of inflammatory triggers, such as lipopolysaccharides (LPS), into the systemic circulation. The intestinal barrier comprises not only the TJs between epithelial cells but also the mucus layer overlaying the epithelium. Research has demonstrated that *Hylocereus polyrhizus* Pulp Residues Polysaccharide can repair high-fat diet-induced damage to the mucus layer by regulating the O-glycosylation of intestinal core mucin, thereby restoring its normal structural and functional integrity (131). Beneath this mucosal layer, the second line of defense consists of the intestinal epithelial cells and their intercellular TJs. Red *Astragalus* polysaccharides have been shown to upregulate the expression of TJ proteins, such as ZO-1 and occludin, in intestinal epithelial cells; this directly repairs the physical intestinal barrier, reduces the influx of inflammatory substances into the systemic circulation, and ultimately protects the BBB from peripheral inflammatory damage (132). Furthermore, phytochemicals can systematically improve the cerebral microenvironment by reshaping the gut microbiota structure, thereby affording BBB protection. Researchers have found that baicalein can precisely inhibit *Prevotella*, a genus positively correlated with neuroinflammation, while simultaneously promoting the growth of *Blautia*, which possesses anti-inflammatory potential. This microbial remodeling reduces upstream inflammatory signaling, creating a low-inflammatory systemic environment conducive to BBB maintenance (133). Similarly, the TCM compound Sanhua Decoction, composed of rhubarb, immature bitter orange, magnolia bark, and notopterygium, can reset the global intestinal environment. It effectively eliminates opportunistic pathogens such as *Escherichia coli* and creates favorable conditions for the proliferation of probiotics, including *Lactobacillus* and *Bifidobacterium* (134). Additionally, phytochemicals can specifically promote the growth of probiotics with potent antioxidant capabilities. A recent study demonstrated that a novel nutritional supplement consisting of rice, silkworm pupa, ginger, and holy basil significantly promoted the proliferation of *Lactobacillus* and *Bifidobacterium*, inhibited oxidative stress levels, indirectly protected the BBB, and ultimately improved post-stroke VCI (135).

The gut microbiota modulates BBB structure and function not only through vagal and immune pathways but also via metabolites—particularly Short-Chain Fatty Acids (SCFAs) such as acetate, propionate, and butyrate. SCFAs can cross the BBB via the circulatory system, enhance TJ protein expression, and inhibit MMP activity, thereby maintaining barrier integrity (136). A study confirmed that phytochemicals can significantly increase the abundance of beneficial *Lactobacillus* and reduce the proportion of the opportunistic pathogen *Escherichia-Shigella* by reshaping the gut microbiota. This optimization of the microbial community directly promotes the production of beneficial SCFAs (acetate, propionate, and butyrate) in the gut and elevates their levels in brain tissue. Crucially, the study revealed that these SCFAs function as signaling molecules to upregulate PPARγ protein expression in the brain, thereby regulating cerebral lipid metabolism. Ultimately, this indirect effect via the “gut-brain” axis inhibits cerebral lipid peroxidation and ferroptosis, exerting a significant neuroprotective effect (137). Additionally, phytochemicals systematically regulate systemic inflammation and oxidative stress by modulating the gut microbiota structure and its metabolic output, thereby improving BBB function. Researchers have suggested that phytochemicals can promote the proliferation of butyrate-producing bacteria and increase circulating butyrate levels (138). Recent studies have found that supplementation with human-derived *Clostridium butyricum* not only increases SCFA levels in the colon and brain tissue but also significantly enhances the expression of the BBB tight junction proteins claudin-5 and occludin while reducing serum levels of LPS and pro-inflammatory cytokines; this exerts a neuroprotective effect and improves cognitive function in a high-fat diet rat model (139).

To provide a comprehensive synthesis of the phytochemicals discussed in this review, Table 1 summarizes their chemical categories, representative compounds or herbs, BBB-targeting mechanisms of action, and the experimental models utilized in these studies. This table highlights the multi-target potential of phytochemicals in ameliorating VCI through diverse pathways, including BBB integrity restoration, anti-inflammatory effects, antioxidant activity, neuroprotection, and gut-brain axis modulation.

**TABLE 1:** Summary of key phytochemicals, their mechanisms of action, and experimental models in targeting BBB for VCI improvement.

Compound category	Representative compound/herb	Mechanism of action	Experimental model
Iridoids	Catalpol	Downregulates RhoA/ROCK2 pathway, repairs tight junction structure (upregulates claudin-5, occludin, ZO-1/2/3), reduces BBB permeability.	Lipopolysaccharide (LPS)-induced BBB injury model
Saponins	*Panax notoginseng* saponins (PNS)	Protects tight junction proteins (ZO-1, claudin-5) from degradation, maintaining BBB structural stability.	Simulated ischemia–reperfusion injury in cerebral microvascular endothelial cells
	Notoginsenoside R1 (NR1)	Inhibits the TLR4/MyD88/NF-κB inflammatory pathway, reducing pro-inflammatory cytokines (TNF-α, IL-1β).	Ischemia-reperfusion injury model
	*Gynostemma pentaphyllum*	Scavenges oxygen free radicals, enhances antioxidant capacity, inhibits astrocyte activation.	Rat model of vascular cognitive impairment
	Bacopaside I	Activates PKC and PI3K/Akt pathways, upregulates p-Akt, reduces neuronal damage.	Cerebral ischemia-induced cognitive deficits model and OGD model
Polyphenols	Resveratrol	Inhibits depolymerization of tight junction proteins (occludin, ZO-1), stabilizes their polymerization state, protects BBB integrity.	High-fat diet-induced BBB injury model
	Grape seed proanthocyanidins	Inhibits MMP-9 activity, restores BBB integrity.	Chemotherapy-induced cognitive impairment (CICI) model
	Quercetin	Protects against ischemic white matter injury, likely via scavenging free radicals.	Rat model of vascular dementia (chronic cerebral hypoperfusion)
	Naringenin	Reduces ROS and MDA, enhances antioxidant enzymes, upregulates NMDA receptor signaling pathway.	Rat model of vascular dementia
Flavonoids	Puerarin	Inhibits TLR4-mediated MyD88-dependent pathway, down-regulates NF-κB, reduces TNF-α.	Rat model of ischemia-reperfusion injury
	Icariin	Enhances phosphorylation of CREB within the central cholinergic circuit, restores histone acetylation homeostasis.	Post-stroke vascular cognitive impairment model
	Baicalein	Regulates gut microbiota (inhibits *Prevotella*, promotes *Blautia*), reduces neuroinflammation.	Vascular dementia rat model
Terpene lactones	XQ-1H (Ginkgolide B derivative)	Inhibits MMP-9 overexpression, protects endothelial ultrastructure, maintains BBB physical barrier.	Animal model of hyperlipidemia combined with ischemic stroke
	Ginkgolide B (GB)	Promotes endothelial cell proliferation, migration, and lumen formation, restoring cerebral blood flow.	OGD/R model and MCAO model
	Ginkgolide C (GC)	Inhibits the CD40/NF-κB signaling pathway, reducing TNF-α and IL-6.	MCAO/R rat model
	Ginkgolide K (GK)	Inhibits GSK-3β-mediated mitochondrial hyperdivision and increased membrane permeability.	MCAO rat model and OGD/R neuron model
Polysaccharides	*Lycium barbarum* polysaccharides	Inhibit overactivation of microglia and astrocytes, suppress P65 NF-κB and P38 MAPK pathways.	MCAO-induced focal cerebral ischemic injury mouse model
	*Astragalus* polysaccharides	Activate the Nrf2-ARE pathway, restore SOD and GSH-Px activities, attenuate oxidative stress.	APP/PS1 mouse model
	*Polygonatum* polysaccharides (POP)	Activate Nrf2 via the Nrf2-ferroptosis pathway, downregulating ferroptosis-related proteins.	D-galactose-induced aging rat model
	Red *Astragalus* polysaccharides (RHP)	Upregulate tight junction proteins (ZO-1, occludin) in intestinal epithelial cells, repairing the intestinal physical barrier.	Senescence-Accelerated Mouse-Prone 8 (*SAMP8*)
Bioactive peptides	Walnut-derived peptide (TWLPLPR)	Down-regulates MMP-9 gene expression and enzyme activity by inhibiting the NF-κB p65/iNos signaling pathway.	(Specific model not mentioned in text) Aβ25-35-injured bEnd.3 cells
	Soy-derived peptide (VHVV)	Up-regulates BDNF, activates CREB and PI3K-AKT-mTOR pathways, promoting neuronal survival.	Hypertension-mediated vascular cognitive impairment model
Other	*Salvia miltiorrhiza* (Danshen)	Inhibits ferroptosis, alleviating ischemic neuronal injury.	Transient middle cerebral artery occlusion (tMCAO) model
	Paeonol	Reduces TUNEL-positive cells, downregulates Bax, suppresses apoptosis-inducing factor (AIF) release.	Rat model of transient middle cerebral artery occlusion (tMCAO)
	Sanhua decoction (TCM formula)	Regulates gut microbiota (eliminates *Escherichia coli*, promotes *Lactobacillus* and *Bifidobacterium*).	Ischemia-reperfusion rat model (via component study)

## 4 Challenges, limitations

### 4.1 Clinical research issues of phytochemicals

Although accumulated preclinical evidence convincingly demonstrates the multi-target potential of phytochemicals in maintaining BBB integrity and ameliorating VCI pathology, a significant translational gap remains between these promising experimental findings and established clinical efficacy (140, 141). This discrepancy is primarily attributable to the paucity of robust clinical research. The majority of discussed mechanisms have been verified primarily in animal models or *in vitro* systems. Current clinical evidence is largely derived from epidemiological associations or small-scale trials (142, 143). The design of randomized controlled trials (RCTs) faces substantial challenges: the etiological complexity of VCI complicates patient selection (144), and variations in plant extract composition impede dosage standardization (145). Furthermore, current research lacks mediating endpoints capable of directly and sensitively reflecting BBB function and mechanisms. Over-reliance on macroscopic neuropsychological scales often fails to capture the precise pharmacological effects on the BBB and the neurovascular unit (146).

### 4.2 Differences in responses of phytochemicals to different VCI subtypes

The etiological heterogeneity of VCI (spanning stroke, hypertension, diabetes mellitus, aging, etc.) dictates the substantial diversity of the patient population (147). However, the majority of clinical and basic studies have failed to conduct large-scale stratified analyses targeting VCI subtypes, potentially obscuring the differential efficacy of phytochemicals. For instance, post-stroke VCI is typically precipitated by acute ischemic events, accompanied by profound neuroinflammation, acute BBB disruption, and rapid MMP-9 upregulation (148). In contrast, hypertension-related VCI is characterized predominantly by chronic vascular injury, oxidative stress, and microvascular remodeling (149). These pathophysiological disparities likely result in distinct therapeutic responses and mechanisms of action for phytochemicals across different subtypes. In post-stroke VCI, Gotu Kola Extract has demonstrated superior efficacy compared to folic acid in improving cognitive outcomes (55). A recent study on renovascular hypertensive rats indicated that the phytochemical carvacrol effectively ameliorates cognitive impairment by reducing blood pressure and inhibiting pro-inflammatory cytokines in the hippocampus and cerebral cortex (150). While some studies have explored the benefits of specific phytochemicals in VCI models, most rely on single animal models that fail to fully recapitulate specific clinical VCI subtypes. Current research remains largely focused on mono-etiological disease models, lacking head-to-head comparisons between different subtypes. This impedes the determination of whether a specific phytochemical confers advantageous effects for a particular subtype (e.g., post-stroke VCI versus hypertension-related VCI). Future preclinical research should prioritize comparative pharmacological studies utilizing animal models that better simulate distinct VCI subtypes. Concurrently, clinical studies must incorporate VCI subtype stratification as a pivotal design factor, employing increasingly precise neuroimaging biomarkers—such as MRI-based radiomic features (151)—and clinical characteristics to finely classify patient populations, thereby revealing optimal phytochemical application strategies for specific VCI subgroups.

### 4.3 The dilemma of phytochemical bioavailability and BBB penetration efficiency

While numerous studies have indicated that phytochemicals hold tremendous promise for improving VCI by targeting the BBB, their translation into human clinical trials has been repeatedly hindered. A critical bottleneck lies in the generally poor systemic bioavailability of these plant-derived nutrients (152). This limitation stems from a series of physiological and biochemical barriers. Following oral administration, phytochemicals undergo complex biotransformations before reaching the systemic circulation and ultimately acting on targets within the CNS, resulting in actual exposure levels that are far below effective therapeutic concentrations. First, upon oral administration, phytochemicals enter the liver via the portal vein, where they are extensively metabolized by Phase I and Phase II enzymes; this conversion into inactive metabolites significantly reduces the plasma concentration of the parent compound (153). Second, certain phytochemicals possess unstable chemical structures within the acidic environment of the gastrointestinal tract, rendering them prone to degradation and loss of efficacy. For instance, daidzein exhibits a relatively low intestinal absorption rate within the gastrointestinal tract (154). Furthermore, many hydrophobic phytochemicals demonstrate extremely low solubility in aqueous environments, resulting in inefficient dissolution from formulations and poor absorption across the intestinal mucosa. For example, apigenin is a bioactive flavonoid with significant therapeutic potential, yet its poor water solubility severely limits its bioavailability (155). Beyond metabolism and absorption, active efflux transport systems present another substantial barrier. Highly expressed efflux transporters on intestinal epithelial cells and capillary endothelial cells, such as P-gp, can actively pump absorbed phytochemicals back into the intestinal lumen or blood, further restricting their bioavailability (156). Even if these compounds withstand the challenges of digestive tract absorption and systemic circulation, therapeutic agents for CNS disorders like VCI must still negotiate the selective filtration of the BBB. The BBB strictly regulates the influx and efflux of substances to maintain CNS homeostasis. According to Lipinski’s Rule of Five, ideal CNS drugs typically possess low molecular weight, moderate lipophilicity, and a limited number of hydrogen bond donors or acceptors. Many phytochemicals fail to meet these criteria due to excessive molecular weight or extreme hydrophilicity or lipophilicity. Consequently, more than 98% of small-molecule drugs and nearly all large-molecule drugs are unable to cross the BBB. Similar to gastrointestinal cells, multidrug resistance proteins such as P-gp and Breast Cancer Resistance Protein, which are highly expressed on BBB endothelial cells, effectively recognize and pump hydrophobic exogenous substances back into the bloodstream, preventing their entry into the brain (157). Additionally, many phytochemicals in the circulation exhibit high binding affinity for plasma proteins such as albumin. Only free, unbound fractions can diffuse across the BBB, which drastically reduces the concentration of drug available for CNS penetration. A representative example is quercetin, which demonstrates a plasma protein binding rate as high as 99.1%—primarily to albumin—resulting in a free concentration of less than 1% (158).

## 5 Future research directions

### 5.1 Targeted delivery systems

To address challenges such as poor water solubility, insufficient targeting, and low bioavailability associated with phytochemicals and active components of TCM in the prevention and treatment of CVD and cognitive impairment, researchers have developed diverse nano-delivery systems that significantly enhance therapeutic efficacy and targeting specificity. Specifically, regarding the application of Ginkgolide B, brain-targeted GB-modified carbonized polymer dots (GB-CPDs) demonstrated excellent BBB penetration capability in a MCAO/R mouse model following tail vein injection. GB-CPDs specifically accumulated within the ischemic penumbra, significantly reducing cerebral infarct volume, improving neurological function, and alleviating cerebral edema (*p* < 0.01). The underlying mechanism involves the inhibition of oxidative stress, neuronal apoptosis, and microglial activation, alongside a reduced release of pro-inflammatory cytokines such as TNF-α and IL-1β, thereby exerting potent neuroprotective effects (159). Similarly, to address the poor water solubility and limited targeting specificity of GB, researchers have developed GB-loaded liposomes capable of selectively targeting the ischemic hemisphere. These liposomes preferentially accumulated in the ischemic cerebral hemisphere of MCAO/R model mice. Compared to free GB, the liposomal formulation more effectively reduced cerebral infarct volume, improved neurological function, and alleviated oxidative stress and inflammatory responses (*p* < 0.01) (160). In the context of resveratrol (Res) delivery, flower-like Res-loaded selenium nanoparticles/chitosan nanoparticles (Res@SeNPs@Res-CS-NPs) were synthesized, exhibiting a high drug-loading capacity of 64%. This delivery system restored gut microbiota homeostasis by increasing the abundance of Bacteroidetes and modulating specific genera such as *Enterococcus*. These changes, in turn, attenuated LPS-induced neuroinflammation, ameliorated lipid deposition and insulin resistance, and consequently improved cognitive function in mice with metabolic disorders (161). To address the challenge of limited drug delivery to the ischemic region in stroke, researchers leveraged the inherent ability of macrophage membranes to penetrate the BBB. They conjugated angelica polysaccharide and ethyl ferulate via an oxalate bond and encapsulated tetramethylpyrazine within the core. The resulting macrophage membrane-camouflaged amphiphilic nanoparticle, MAOE@TMP, facilitated targeted drug delivery and specific release at the brain injury site, providing neuroprotective effects, scavenging reactive oxygen species, and exerting anti-inflammatory activity. Compared with the clinical drug tetramethylpyrazine hydrochloride, MAOE@TMP delivered drugs more efficiently and significantly reduced cerebral infarct volume (162). In the exploration of chlorogenic acid (CGA), brain-targeting peptide-modified flower-like selenium nanoclusters (TGN-CGA@SeNCs) successfully overcame the limitation of low CGA bioavailability by enhancing its solubility and stability (163). Additionally, targeting the poor water solubility and low bioavailability of herbal borneol, a TPGS-g-guar-gum nanoparticle drug delivery system was developed by combining vitamin E d-α-tocopherol polyethylene glycol succinate (TPGS) and guar gum. This system significantly improved the solubility and drug-loading capacity of borneol, achieving effective encapsulation and release (164). These nano-delivery systems provide efficient strategies for the application of phytochemicals and active TCM ingredients in the prevention and treatment of CVD and cognitive impairments by optimizing drug targeting, stability, and bioavailability.

In recent years, significant advancements have been achieved in neuroscience regarding the utilization of exosomes as brain-targeted delivery platforms. As endogenous nanovesicles, exosomes are considered ideal carriers for cerebral delivery due to their innate biocompatibility, low immunogenicity, and intrinsic capacity to traverse the BBB. A study investigating plant-derived exosomes confirmed that small extracellular vesicles extracted from *Momordica charantia* (MC-sEVs) can cross the BBB, accumulate within the brain, significantly alleviate neuronal ferroptosis, and promote neurological functional recovery in an ischemic stroke model. The underlying mechanism involves miR-5813b—a plant-specific microRNA enriched in MC-sEVs—which directly targets the E3 ubiquitin ligase TRIM62, thereby preserving neuronal survival under ischemic stress (165). Another study focusing on extracellular vesicle-like particles derived from *Houttuynia cordata* Thunb (HT-EVLP) corroborated the potential of plant-derived vesicles in VCI intervention through a distinct pathway. HT-EVLP exhibits excellent BBB penetration capability, specifically accumulating in cerebral ischemic regions to significantly alleviate neuronal damage and promote neurological recovery. Its unique mechanism of action is mediated by miR159a, a plant miRNA enriched in HT-EVLP that directly targets and inhibits the expression of long-chain acyl-CoA synthetase 4 (ACSL4). ACSL4 is a key enzyme in lipid metabolism responsible for catalyzing the esterification of polyunsaturated fatty acids that drive ferroptosis. Consequently, through this mechanism, HT-EVLP effectively inhibits ferroptosis at the “upstream” source: the biosynthetic generation of lipid peroxidation substrates (166). The focus of advanced research has shifted from natural exosomes to engineered exosomes, aiming to enhance targeting specificity and therapeutic efficacy through functional modification or structural fusion. For instance, Xie et al. (167) developed a hybrid nanosystem (Exo–Lip) by fusing neural stem cell-derived exosomes with liposomes loaded with TCM extracts. This system exhibited excellent BBB penetration capacity and exerted dual regulatory effects on neuroinflammation and lipid metabolism in ischemic stroke models. This bioengineering strategy not only improves exosome stability and drug delivery efficiency but also offers novel perspectives for treating complex cerebrovascular diseases. Although relevant studies in the specific field of VCI remain limited, the neuroprotective and metabolic regulatory mechanisms demonstrated by Exo–Lip suggest significant potential for application in VaD (167).

Notably, beyond traditional nanocarrier modification, molecular-level targeting tools exemplified by peptides are demonstrating superior precision and application potential. Possessing advantages such as low molecular weight, low immunogenicity, and ease of synthesis and functional modification, peptides serve as ideal “navigation moieties” anchored to nanocarriers or drug molecules, enabling precise BBB traversal via active targeting mechanisms (168). A novel peptide-modified liposomal system developed by Chen et al. (169) utilized ROS-responsive liposomes modified with the RVG29 targeting peptide (PUELipo/R-R) for puerarin delivery. This peptide specifically recognizes nAChR on the surface of BBB endothelial cells and neurons, mediating receptor-mediated endocytosis to achieve precise cerebral drug delivery. This peptide modification strategy not only effectively surmounts the BBB barrier but also significantly enhances the accumulation of nanocarriers within ischemic regions (169). Similarly, Yang et al. (170) surface-modified gelatin nanoparticles with the RVG29 peptide. By leveraging the specific binding affinity between RVG29 and nAChR receptors on the BBB and neurons, this approach achieved efficient intracerebral delivery of curcumin-loaded gelatin nanoparticles (Cur@Gel NPs). This targeting strategy aligns with the findings of Chen et al. (169) regarding RVG29-modified liposomes, collectively confirming that peptide-mediated receptor targeting represents a versatile and efficient core technology for overcoming the BBB and achieving precise intracerebral drug accumulation. Crucially, Yang’s study further verified that, attributable to RVG29 modification, the targeted nanocarrier (Cur@GAR NPs) exhibited superior improvement in neurobehavioral function compared to unmodified nanoparticles (Cur@Gel NPs) in cerebral ischemia models. This underscores the decisive contribution of precise targeting to the ultimate therapeutic outcome. Consequently, peptide-modified nanoplatforms, such as those utilizing RVG29, have established a solid technical foundation for the development of therapies capable of directly intervening in the central pathological processes of VCI (170).

### 5.2 Targeted clinical trial design

Future clinical research is expected to pivot toward mechanism-informed clinical trials that utilize biomarker enrichment strategies. For instance, establishing MRI-confirmed BBB disruption as an inclusion criterion can enrich patient cohorts with defined vascular pathologies, thereby enhancing the homogeneity and targeted nature of the study. Furthermore, the integration of multi-sequence MRI radiomics features with clinical variables to construct predictive models facilitates the accurate identification of VCI associated with BBB injury (151). Building upon this, patient stratification can be further refined by incorporating resting-state functional magnetic resonance imaging (rs-fMRI) metrics, such as ALFF, ReHo, and FC. An Activation Likelihood Estimation meta-analysis revealed that patients with VCI consistently exhibit reduced ALFF and diminished FC at key nodes of the default mode network, specifically the precuneus and cingulate gyrus. Given that these regions are particularly sensitive to hypoperfusion and vascular injury, combining BBB disruption data with specific functional network abnormalities allows for the precise identification of VCI subtypes sharing similar pathophysiological phenotypes. Consequently, future radiomics research in VCI should actively integrate multimodal neuroimaging and clinical variables to construct comprehensive models linking BBB integrity with brain function (146). Clinical trials must also integrate biomarkers capable of reflecting drug target engagement and downstream biological effects. For example, measuring the dynamic changes in plasma MMP-9 levels, inflammatory cytokines, and oxidative stress markers (such as MDA) before and after phytochemical intervention is essential. These objective biological indicators provide direct human-level evidence for mechanisms elucidated in preclinical studies and may be detectable prior to macroscopic improvements in cognitive function. Recent evidence indicates that plasma neurofilament light chain (NfL) and glial fibrillary acidic protein (GFAP) can effectively distinguish VCI from non-vascular cognitive etiologies. This suggests that stratifying patients based on baseline plasma biomarker levels can enhance the sensitivity and specificity of clinical trials, thereby aiding in the identification of subpopulations most likely to benefit from intervention (171). Large-scale clinical studies have systematically investigated circulating markers reflecting BBB dysfunction and vascular pathology. Research confirms that members of the vascular endothelial growth factor family, such as placental growth factor (PLGF) and VEGF-D, are significantly associated with WMH volume as well as plasma markers of neuroinflammation (GFAP) and neurodegeneration (NfL). Consequently, monitoring dynamic changes in vascular-related biomarkers like PLGF can provide direct clinical evidence regarding the pathological mechanisms underlying vascular-derived cognitive impairment (172). Given the extreme difficulty of reversing neuronal damage once dementia is established, the most pragmatic near-term clinical scenario involves early intervention and primary prevention in high-risk populations. It is imperative to conduct large-scale, long-term, prospective RCTs involving individuals with vascular risk factors, such as hypertension and diabetes, to determine whether standardized and clearly defined phytochemical supplementation can delay or prevent the onset and progression of VCI. Such efforts will transform the protective associations observed in epidemiological studies into evidence-based, actionable prevention strategies (173).

## 6 Conclusion

This review systematically summarizes and synthesizes the potential of phytochemicals to ameliorate VCI through multiple mechanisms targeting the BBB, including the restoration of TJs, inhibition of MMP activity, modulation of neuroinflammation and oxidative stress, promotion of functional recovery in the neurovascular unit, and regulation of the gut–brain axis. These mechanisms work synergistically to highlight the unique advantages of phytochemicals as a multi-target intervention strategy, particularly for complex diseases like VCI that involve multifactorial etiologies and diverse pathological pathways. However, current research presents evident limitations. First, the majority of evidence is derived from animal models or *in vitro* experiments, whereas clinical research remains relatively scarce and limited in scale. Second, inherent challenges associated with phytochemicals, such as low bioavailability and poor BBB penetration, restrict their clinical translation. Future studies are recommended to prioritize the conduct of high-quality clinical trials to validate the efficacy of specific phytochemicals or compound formulations in VCI populations. Additionally, strategies such as nanotechnology and brain-targeted delivery systems are expected to be leveraged to optimize intracerebral delivery efficiency and therapeutic precision. Furthermore, comprehensive exploration of the synergistic interactions between phytochemicals and conventional pharmacotherapies, as well as their differential effects across various VCI subtypes and disease stages, is essential to provide a scientific basis for the development of personalized nutritional intervention strategies.

## References

[1] Rundek T Tolea M Ariko T Fagerli E Camargo C. Vascular cognitive impairment (VCI). *Neurotherapeutics.* (2022) 19:68–88. 10.1007/s13311-021-01170-y 34939171 PMC9130444

[2] Munthe-Kaas R Aam S Ihle-Hansen H Lydersen S Knapskog A Wyller T Impact of different methods defining post-stroke neurocognitive disorder: the Nor-COAST study. *Alzheimers Dement.* (2020) 6:e12000. 10.1002/trc2.12000 32211505 PMC7085256

[3] He A Wang Z Wu X Sun W Yang K Feng W Incidence of post-stroke cognitive impairment in patients with first-ever ischemic stroke: a multicenter cross-sectional study in China. *Lancet Reg Health West Pac.* (2023) 33:100687. 10.1016/j.lanwpc.2023.100687 37181529 PMC10166998

[4] Kalaria R. The pathology and pathophysiology of vascular dementia. *Neuropharmacology.* (2018) 134:226–39. 10.1016/j.neuropharm.2017.12.030 29273521

[5] Wolters F Ikram M. Epidemiology of vascular dementia. *Arteriosclerosis Thrombosis Vascular Biol.* (2019) 39:1542–9. 10.1161/ATVBAHA.119.311908 31294622

[6] O’Brien J Thomas A. Vascular dementia. *Lancet.* (2015) 386:1698–706. 10.1016/S0140-6736(15)00463-8 26595643

[7] Obermeier B Daneman R Ransohoff R. Development, maintenance and disruption of the blood-brain barrier. *Nat Med.* (2013) 19:1584–96. 10.1038/nm.3407 24309662 PMC4080800

[8] Haseloff R Dithmer S Winkler L Wolburg H Blasig I. Transmembrane proteins of the tight junctions at the blood-brain barrier: structural and functional aspects. *Semin Cell Dev Biol.* (2015) 38:16–25. 10.1016/j.semcdb.2014.11.004 25433243

[9] Otani T Furuse M. Tight junction structure and function revisited. *Trends Cell Biol.* (2020) 30:805–17. 10.1016/j.tcb.2020.08.004 32891490

[10] Garbuzova-Davis S Mirtyl S Sallot S Hernandez-Ontiveros D Haller E Sanberg P. Blood-brain barrier impairment in MPS III patients. *BMC Neurol.* (2013) 13:174. 10.1186/1471-2377-13-174 24225396 PMC3835134

[11] Rajeev V Fann D Dinh Q Kim H De Silva T Lai M Pathophysiology of blood brain barrier dysfunction during chronic cerebral hypoperfusion in vascular cognitive impairment. *Theranostics.* (2022) 12:1639–58. 10.7150/thno.68304 35198062 PMC8825579

[12] Abdullahi W Tripathi D Ronaldson P. Blood-brain barrier dysfunction in ischemic stroke: targeting tight junctions and transporters for vascular protection. *Am J Physiol Cell Physiol.* (2018) 315:C343–56. 10.1152/ajpcell.00095.2018 29949404 PMC6171039

[13] Wang T Liu Y Xu X Deng C Wu K Zhu J Lgl1 activation of rab10 promotes axonal membrane trafficking underlying neuronal polarization. *Dev Cell.* (2011) 21:431–44. 10.1016/j.devcel.2011.07.007 21856246

[14] Hall C Reynell C Gesslein B Hamilton N Mishra A Sutherland B Capillary pericytes regulate cerebral blood flow in health and disease. *Nature.* (2014) 508:55–60. 10.1038/nature13165 24670647 PMC3976267

[15] Rudziak P Ellis C Kowalewska P. Role and molecular mechanisms of pericytes in regulation of leukocyte diapedesis in inflamed tissues. *Mediators Inflamm.* (2019) 2019:4123605. 10.1155/2019/4123605 31205449 PMC6530229

[16] Halder S Sapkota A Milner R. The impact of genetic manipulation of laminin and integrins at the blood-brain barrier. *Fluids Barriers CNS.* (2022) 19:50. 10.1186/s12987-022-00346-8 35690759 PMC9188059

[17] Li T Zheng J Wang Z Xu L Sun D Song H Maresin 1 improves cognitive decline and ameliorates inflammation and blood-brain barrier damage in rats with chronic cerebral hypoperfusion. *Brain Res.* (2022) 1788:147936. 10.1016/j.brainres.2022.147936 35533741

[18] Cao Q Chen J Zhang Z Shu S Qian Y Yang L Astrocytic CXCL5 hinders microglial phagocytosis of myelin debris and aggravates white matter injury in chronic cerebral ischemia. *J Neuroinflammation.* (2023) 20:105. 10.1186/s12974-023-02780-3 37138312 PMC10155379

[19] Zhang Y Zou Z Li R Fu X Li G Wang L DTX1 modulates microglial M1 polarization and exacerbates neuroinflammation in traumatic brain injury model rats through NF-κB/IRF5. *Mol Neurobiol.* (2025) 62:14140–55. 10.1007/s12035-025-05200-0 40660011 PMC12511207

[20] Wu Q Jiang N Wang Y Song G Li P Fang Y Soluble epoxide hydrolase inhibitor (TPPU) alleviates ferroptosis by regulating CCL5 after intracerebral hemorrhage in mice. *Biomed Pharmacother.* (2024) 172:116301. 10.1016/j.biopha.2024.116301 38377737

[21] Wang L Mao B Fan K Sun R Zhang J Liang H attenuates TET2-dependent ZO-1 epigenetic expression in cerebral vascular endothelial cells. *Fluids Barriers CNS.* (2022) 19:73. 10.1186/s12987-022-00370-8 36076297 PMC9461112

[22] Bauer A Bürgers H Rabie T Marti H. Matrix metalloproteinase-9 mediates hypoxia-induced vascular leakage in the brain via tight junction rearrangement. *J Cereb Blood Flow Metab.* (2010) 30:837–48. 10.1038/jcbfm.2009.248 19997118 PMC2949161

[23] Debette S Beiser A DeCarli C Au R Himali J Kelly-Hayes M Association of MRI markers of vascular brain injury with incident stroke, mild cognitive impairment, dementia, and mortality: the Framingham offspring study. *Stroke.* (2010) 41:600–6. 10.1161/STROKEAHA.109.570044 20167919 PMC2847685

[24] Taheri S Gasparovic C Huisa B Adair J Edmonds E Prestopnik J Blood-brain barrier permeability abnormalities in vascular cognitive impairment. *Stroke.* (2011) 42:2158–63. 10.1161/STROKEAHA.110.611731 21719768 PMC3584170

[25] Ueno M Chiba Y Murakami R Matsumoto K Fujihara R Uemura N Disturbance of intracerebral fluid clearance and blood-brain barrier in vascular cognitive impairment. *Int J Mol Sci.* (2019) 20:2600. 10.3390/ijms20102600 31137875 PMC6566824

[26] Toyama K Spin J Tsao P. Role of microRNAs on blood brain barrier dysfunction in vascular cognitive impairment. *Curr Drug Deliv.* (2017) 14:744–57. 10.2174/1567201813666160830124627 27572324

[27] Seo J Miyamoto N Hayakawa K Pham L Maki T Ayata C Oligodendrocyte precursors induce early blood-brain barrier opening after white matter injury. *J Clin Invest.* (2013) 123:782–6. 10.1172/JCI65863 23281396 PMC3561802

[28] Wallin A Sjögren M Edman A Blennow K Regland B. Symptoms, vascular risk factors and blood-brain barrier function in relation to CT white-matter changes in dementia. *Eur Neurol.* (2000) 44:229–35. 10.1159/000008242 11096223

[29] Li M Li Y Zuo L Hu W Jiang T. Increase of blood-brain barrier leakage is related to cognitive decline in vascular mild cognitive impairment. *BMC Neurol.* (2021) 21:159. 10.1186/s12883-021-02189-6 33858381 PMC8048027

[30] Iametti S Bordoni A Di Nunzio M. Formation of plant derived bioactive peptides during simulated gastro-intestinal digestion: a systematic review. *Biofactors.* (2025) 51:e70043. 10.1002/biof.70043 40838664

[31] Li C Zhang L Li X Hu Q Mao L Shao Y Sulforaphane suppresses Aβ accumulation and tau hyperphosphorylation in vascular cognitive impairment(VCI). *J Nutr Biochem.* (2025) 136:109803. 10.1016/j.jnutbio.2024.109803 39551165

[32] do Rosario V Fitzgerald Z Broyd S Paterson A Roodenrys S Thomas S Food anthocyanins decrease concentrations of TNF-α in older adults with mild cognitive impairment: a randomized, controlled, double blind clinical trial. *Nutr Metab Cardiovasc Dis.* (2021) 31:950–60. 10.1016/j.numecd.2020.11.024 33546942

[33] Anggraini D Ilyas S Hasibuan P Machrina Y Widyawati T Rusdiana R The potential of Andaliman (Zanthoxylum acanthopodium DC) fruit as an ethanol extract for neuroprotection in aged model rat. *J Adv Vet Anim Res.* (2023) 10:587–92. 10.5455/javar.2023.j713 38370899 PMC10868695

[34] Li W Sun L Yue L Li G Xiao S. The association between eating green vegetables every day and mild cognitive impairment: a community-based cross-sectional study in Shanghai. *Neuropsychiatr Dis Treat.* (2019) 15:3213–8. 10.2147/NDT.S221074 31819449 PMC6875499

[35] Sheng L Jiang Y Alperet D Feng L Pan A Koh W. Quantity and variety of fruit and vegetable intake in midlife and cognitive impairment in late life: a prospective cohort study. *Br J Nutr.* (2023) 129:2084–93. 10.1017/S0007114522000848 35282850

[36] Mottaghi T Amirabdollahian F Haghighatdoost F. Fruit and vegetable intake and cognitive impairment: a systematic review and meta-analysis of observational studies. *Eur J Clin Nutr.* (2018) 72:1336–44. 10.1038/s41430-017-0005-x 29235561

[37] An R Liu G Khan N Yan H Wang Y. Dietary habits and cognitive impairment risk among oldest-old Chinese. *J Gerontol B Psychol Sci Soc Sci.* (2019) 74:474–83. 10.1093/geronb/gbw170 28184889

[38] Zhou Y Wang J Cao L Shi M Liu H Zhao Y Fruit and vegetable consumption and cognitive disorders in older adults: a meta-analysis of observational studies. *Front Nutr.* (2022) 9:871061. 10.3389/fnut.2022.871061 35795585 PMC9251442

[39] Nakamoto M Otsuka R Nishita Y Tange C Tomida M Kato Y Soy food and isoflavone intake reduces the risk of cognitive impairment in elderly Japanese women. *Eur J Clin Nutr.* (2018) 72:1458–62. 10.1038/s41430-017-0061-2 29348624

[40] Kaddoumi A Denney T Deshpande G Robinson J Beyers R Redden D Extra-virgin olive oil enhances the blood-brain barrier function in mild cognitive impairment: a randomized controlled trial. *Nutrients.* (2022) 14:5102. 10.3390/nu14235102 36501136 PMC9736478

[41] GBD 2021 Stroke Risk Factor Collaborators. Global, regional, and national burden of stroke and its risk factors, 1990-2021: a systematic analysis for the Global Burden of Disease Study 2021. *Lancet Neurol.* (2024) 23:973–1003. 10.1016/S1474-4422(24)00369-7 39304265 PMC12254192

[42] Ben Assayag E Korczyn A Giladi N Goldbourt U Berliner A Shenhar-Tsarfaty S Predictors for poststroke outcomes: the Tel aviv brain acute stroke cohort (TABASCO) study protocol. *Int J Stroke.* (2012) 7:341–7. 10.1111/j.1747-4949.2011.00652.x 22044517

[43] Ma F Li L Xu L Wu J Zhang A Liao J The relationship between systemic inflammation index, systemic immune-inflammatory index, and inflammatory prognostic index and 90-day outcomes in acute ischemic stroke patients treated with intravenous thrombolysis. *J Neuroinflammation.* (2023) 20:220. 10.1186/s12974-023-02890-y 37777768 PMC10543872

[44] Zhang A Zhu Y Liao J Wu D Yan X Chen J The association of systemic inflammatory response index and neutrophil-to-high-density lipoprotein ratio mediated by fasting blood glucose with 90-day prognosis in acute ischemic stroke patients. *Neuroepidemiology.* (2025) 59:31–42. 10.1159/000539132 38749405 PMC11797957

[45] Zhu Y Xue G Xu S Qin Q Liu P Ji L Shaped relationship of serum albumin and neurological functional outcomes after acute ischemic stroke: a prospective cohort study. *Neurol Ther.* (2025) 14:949–64. 10.1007/s40120-025-00729-7 40237930 PMC12089567

[46] Sun J Tan L Yu J. Post-stroke cognitive impairment: epidemiology, mechanisms and management. *Ann Transl Med.* (2014) 2:80. 10.3978/j.issn.2305-5839.2014.08.05 25333055 PMC4200648

[47] Oveisgharan S Dawe R Yu L Kapasi A Arfanakis K Hachinski V Frequency and underlying pathology of pure vascular cognitive impairment. *JAMA Neurol.* (2022) 79:1277–86. 10.1001/jamaneurol.2022.3472 36279115 PMC9593318

[48] Kalaria R Akinyemi R Ihara M. Stroke injury, cognitive impairment and vascular dementia. *Biochim Biophys Acta.* (2016) 1862:915–25. 10.1016/j.bbadis.2016.01.015 26806700 PMC4827373

[49] Pohjasvaara T Mäntylä R Salonen O Aronen H Ylikoski R Hietanen M How complex interactions of ischemic brain infarcts, white matter lesions, and atrophy relate to poststroke dementia. *Arch Neurol.* (2000) 57:1295–300. 10.1001/archneur.57.9.1295 10987896

[50] Chen A Akinyemi R Hase Y Firbank M Ndung’u M Foster V Frontal white matter hyperintensities, clasmatodendrosis and gliovascular abnormalities in ageing and post-stroke dementia. *Brain.* (2016) 139:242–58. 10.1093/brain/awv328 26667280 PMC4905522

[51] Giraud M Cho T Nighoghossian N Maucort-Boulch D Deiana G Østergaard L Early blood brain barrier changes in acute ischemic stroke: a sequential MRI study. *J Neuroimaging.* (2015) 25:959–63. 10.1111/jon.12225 25702824

[52] Candelario-Jalil E Dijkhuizen R Magnus T. Neuroinflammation, stroke, blood-brain barrier dysfunction, and imaging modalities. *Stroke.* (2022) 53:1473–86. 10.1161/STROKEAHA.122.036946 35387495 PMC9038693

[53] Jiang X Andjelkovic A Zhu L Yang T Bennett M Chen J Blood-brain barrier dysfunction and recovery after ischemic stroke. *Prog Neurobiol.* (2018) 163–164:144–71. 10.1016/j.pneurobio.2017.10.001 28987927 PMC5886838

[54] Zhang Y Yang H Li S Li W Wang Y. Consumption of coffee and tea and risk of developing stroke, dementia, and poststroke dementia: a cohort study in the UK Biobank. *PLoS Med.* (2021) 18:e1003830. 10.1371/journal.pmed.1003830 34784347 PMC8594796

[55] Farhana K Malueka R Wibowo S Gofir A. Effectiveness of gotu kola extract 750 mg and 1000 mg compared with folic acid 3 mg in improving vascular cognitive impairment after stroke. *Evid Based Complement Alternat Med.* (2016) 2016:2795915. 10.1155/2016/2795915 27340413 PMC4908235

[56] Forette F Seux M Staessen J Thijs L Birkenhäger W Babarskiene M Prevention of dementia in randomised double-blind placebo-controlled systolic hypertension in Europe (Syst-Eur) trial. *Lancet.* (1998) 352:1347–51. 10.1016/s0140-6736(98)03086-4 9802273

[57] Santisteban M Iadecola C Carnevale D. Hypertension, neurovascular dysfunction, and cognitive impairment. *Hypertension.* (2023) 80:22–34. 10.1161/HYPERTENSIONAHA.122.18085 36129176 PMC9742151

[58] Qiu C Winblad B Fratiglioni L. The age-dependent relation of blood pressure to cognitive function and dementia. *Lancet Neurol.* (2005) 4:487–99. 10.1016/S1474-4422(05)70141-1 16033691

[59] König M Palmer K Malsch C Steinhagen-Thiessen E Demuth I. Polyvascular atherosclerosis and renal dysfunction increase the odds of cognitive impairment in vascular disease: findings of the LipidCardio study. *Eur J Med Res.* (2024) 29:141. 10.1186/s40001-024-01734-6 38388510 PMC10882759

[60] van den Kerkhof M de Jong J Voorter P Postma A Kroon A van Oostenbrugge R Blood-brain barrier integrity decreases with higher blood pressure: a 7T DCE-MRI study. *Hypertension.* (2024) 81:2162–72. 10.1161/HYPERTENSIONAHA.123.22617 39136128 PMC11404763

[61] Zhang Z Zhao L Zhou X Meng X Zhou X. Role of inflammation, immunity, and oxidative stress in hypertension: new insights and potential therapeutic targets. *Front Immunol.* (2022) 13:1098725. 10.3389/fimmu.2022.1098725 36703963 PMC9871625

[62] Wang L Liu T Wang X Tong L Chen G Zhou S Microglia-derived TNF-α contributes to RVLM neuronal mitochondrial dysfunction via blocking the AMPK-Sirt3 pathway in stress-induced hypertension. *J Neuroinflammation.* (2023) 20:137. 10.1186/s12974-023-02818-6 37264405 PMC10236846

[63] Boroujerdi A Welser-Alves J Milner R. Matrix metalloproteinase-9 mediates post-hypoxic vascular pruning of cerebral blood vessels by degrading laminin and claudin-5. *Angiogenesis.* (2015) 18:255–64. 10.1007/s10456-015-9464-7 25812799

[64] Ahmadighadykolaei H Lambert J Raeeszadeh-Sarmazdeh M. TIMP-1 protects tight junctions of brain endothelial cells from MMP-mediated degradation. *Pharm Res.* (2023) 40:2121–31. 10.1007/s11095-023-03593-y 37700105 PMC10878538

[65] Takata F Nakagawa S Matsumoto J Dohgu S. Blood-brain barrier dysfunction amplifies the development of neuroinflammation: understanding of cellular events in brain microvascular endothelial cells for prevention and treatment of BBB dysfunction. *Front Cell Neurosci.* (2021) 15:661838. 10.3389/fncel.2021.661838 34588955 PMC8475767

[66] Chen J Vipin A Sandhu G Leow Y Zailan F Tanoto P Blood-brain barrier integrity disruption is associated with both chronic vascular risk factors and white matter hyperintensities. *J Prev Alzheimers Dis.* (2025) 12:100029. 10.1016/j.tjpad.2024.100029 39863325 PMC12184052

[67] Li X Liang J Zheng F. Association between hypertension, diabetes, depression, and serum calcium with the risk of all-cause and vascular dementia: findings from the UK biobank. *Eur J Nutr.* (2024) 64:37. 10.1007/s00394-024-03556-y 39614984

[68] Cevikelli-Yakut Z-A Ertas B Sen A Koyuncuoglu T Yegen BC Sener G. Myrtus communis improves cognitive impairment in renovascular hypertensive rats. *J Physiol Pharmacol.* (2020) 71:665–77. 10.26402/jpp.2020.5.07 33475094

[69] Liu Y Zhao J Han W Yang H Wu X Xie F Microvascular burden and long-term risk of stroke and dementia in type 2 diabetes mellitus. *J Affect Disord.* (2024) 354:68–74. 10.1016/j.jad.2024.03.053 38479499

[70] Yang F Wang Z Zhang J Tang J Liu X Tan L Receptor for advanced glycation end-product antagonist reduces blood-brain barrier damage after intracerebral hemorrhage. *Stroke.* (2015) 46:1328–36. 10.1161/STROKEAHA.114.008336 25782468

[71] Oyama T Miyasita Y Watanabe H Shirai K. The role of polyol pathway in high glucose-induced endothelial cell damages. *Diabetes Res Clin Pract.* (2006) 73:227–34. 10.1016/j.diabres.2006.02.010 16624439

[72] Szabo C. Role of nitrosative stress in the pathogenesis of diabetic vascular dysfunction. *Br J Pharmacol.* (2009) 156:713–27. 10.1111/j.1476-5381.2008.00086.x 19210748 PMC2697759

[73] Wang S Wang J Zhao A Li J. SIRT1 activation inhibits hyperglycemia-induced apoptosis by reducing oxidative stress and mitochondrial dysfunction in human endothelial cells. *Mol Med Rep.* (2017) 16:3331–8. 10.3892/mmr.2017.7027 28765962

[74] Kalani M. The importance of endothelin-1 for microvascular dysfunction in diabetes. *Vasc Health Risk Manag.* (2008) 4:1061–8. 10.2147/vhrm.s3920 19183753 PMC2605330

[75] Shenouda S Widlansky M Chen K Xu G Holbrook M Tabit C Altered mitochondrial dynamics contributes to endothelial dysfunction in diabetes mellitus. *Circulation.* (2011) 124:444–53. 10.1161/CIRCULATIONAHA.110.014506 21747057 PMC3149100

[76] Han J Zhang W Wang Y Cai W Lv G Hu D. Insulin protects against damage to pulmonary endothelial tight junctions after thermal injury: relationship with zonula occludens-1, F-actin, and AKT activity. *Wound Repair Regen.* (2014) 22:77–84. 10.1111/wrr.12128 24393155

[77] Symons J McMillin S Riehle C Tanner J Palionyte M Hillas E Contribution of insulin and Akt1 signaling to endothelial nitric oxide synthase in the regulation of endothelial function and blood pressure. *Circ Res.* (2009) 104:1085–94. 10.1161/CIRCRESAHA.108.189316 19342603 PMC2936913

[78] Lee K Yoon S Hwang I Ma J Yang E Kim R Hyperglycemia enhances brain susceptibility to lipopolysaccharide-induced neuroinflammation via astrocyte reprogramming. *J Neuroinflammation.* (2024) 21:137. 10.1186/s12974-024-03136-1 38802820 PMC11131277

[79] Bahniwal M Little J Klegeris A. High glucose enhances neurotoxicity and inflammatory cytokine secretion by stimulated human astrocytes. *Curr Alzheimer Res.* (2017) 14:731–41. 10.2174/1567205014666170117104053 28124586

[80] de Pins B Cifuentes-Díaz C Farah A López-Molina L Montalban E Sancho-Balsells A Conditional BDNF delivery from astrocytes rescues memory deficits, spine density, and synaptic properties in the 5xFAD mouse model of Alzheimer disease. *J Neurosci.* (2019) 39:2441–58. 10.1523/JNEUROSCI.2121-18.2019 30700530 PMC6435824

[81] Raffaitin C Gin H Empana J Helmer C Berr C Tzourio C Metabolic syndrome and risk for incident Alzheimer’s disease or vascular dementia: the three-city study. *Diabetes Care.* (2009) 32:169–74. 10.2337/dc08-0272 18945929 PMC2606808

[82] Zhan M Liu X Xia X Yang Y Xie Y Zhang L Promotion of neuroinflammation by the glymphatic system: a new insight into ethanol extracts from Alisma orientale in alleviating obesity-associated cognitive impairment. *Phytomedicine.* (2024) 122:155147. 10.1016/j.phymed.2023.155147 37864890

[83] Callewaert B Gsell W Lox M Himmelreich U Jones EAV. A timeline study on vascular co-morbidity induced cerebral endothelial dysfunction assessed by perfusion MRI. *Neurobiol Dis.* (2024) 202:106709. 10.1016/j.nbd.2024.106709 39433136

[84] Tomassoni D Martinelli I Moruzzi M Micioni Di Bonaventura MV Cifani C Amenta F Obesity and age-related changes in the brain of the Zucker Lepr fa/fa Rats. *Nutrients.* (2020) 12:1356. 10.3390/nu12051356 32397542 PMC7284640

[85] Pistell P Morrison C Gupta S Knight A Keller J Ingram D Cognitive impairment following high fat diet consumption is associated with brain inflammation. *J Neuroimmunol.* (2010) 219:25–32. 10.1016/j.jneuroim.2009.11.010 20004026 PMC2823983

[86] Toribio-Mateas M. Harnessing the power of microbiome assessment tools as part of neuroprotective nutrition and lifestyle medicine interventions. *Microorganisms.* (2018) 6:35. 10.3390/microorganisms6020035 29693607 PMC6027349

[87] Csik B Nyúl-Tóth Á Gulej R Patai R Kiss T Delfavero J Senescent endothelial cells in cerebral microcirculation are key drivers of age-related blood-brain barrier disruption, microvascular rarefaction, and neurovascular coupling impairment in mice. *Aging Cell.* (2025) 24:e70048. 10.1111/acel.70048 40167015 PMC12266767

[88] Abdul Y Karakaya E Chandran R Jamil S Ergul A. Endothelin A receptors contribute to senescence of brain microvascular endothelial cells. *Can J Physiol Pharmacol.* (2022) 100:1087–96. 10.1139/cjpp-2022-0071 36384316 PMC10052805

[89] Todorov-Völgyi K González-Gallego J Müller S Beaufort N Malik R Schifferer M Proteomics of mouse brain endothelium uncovers dysregulation of vesicular transport pathways during aging. *Nat Aging.* (2024) 4:595–612. 10.1038/s43587-024-00598-z 38519806

[90] Bell R Winkler E Singh I Sagare A Deane R Wu Z Apolipoprotein E controls cerebrovascular integrity via cyclophilin A. *Nature.* (2012) 485:512–6. 10.1038/nature11087 22622580 PMC4047116

[91] Chen Y Joo J Chu J Chang R Wong G. Downregulation of the glucose transporter GLUT 1 in the cerebral microvasculature contributes to postoperative neurocognitive disorders in aged mice. *J Neuroinflammation.* (2023) 20:237. 10.1186/s12974-023-02905-8 37858199 PMC10588063

[92] Pekcec A Schneider E Baumgärtner W Stein V Tipold A Potschka H. Age-dependent decline of blood-brain barrier P-glycoprotein expression in the canine brain. *Neurobiol Aging.* (2011) 32:1477–85. 10.1016/j.neurobiolaging.2009.08.014 19836857

[93] Bors L Tóth K Tóth E Bajza Á Csorba A Szigeti K Age-dependent changes at the blood-brain barrier. A Comparative structural and functional study in young adult and middle aged rats. *Brain Res Bull.* (2018) 139:269–77. 10.1016/j.brainresbull.2018.03.001 29522862

[94] Zeng L Zou Y Kong L Wang N Wang Q Wang L Can Chinese herbal medicine adjunctive therapy improve outcomes of senile vascular dementia? Systematic review with meta-analysis of clinical trials. *Phytother Res.* (2015) 29:1843–57. 10.1002/ptr.5481 26443194

[95] Fisher N Sorond F Hollenberg N. Cocoa flavanols and brain perfusion. *J Cardiovasc Pharmacol.* (2006) 47:S210–4. 10.1097/00005344-200606001-00017 16794460

[96] Feng C Cao L Luo D Ju L Yang J Xu X Dendrobium polysaccharides attenuate cognitive impairment in senescence-accelerated mouse prone 8 mice via modulation of microglial activation. *Brain Res* (2019) 1704:1–10. 10.1016/j.brainres.2018.09.030 30253123

[97] Tian J Huo R Wang Y Wang J Fang F Fang C. Astragalus polysaccharide alleviates cognitive decline in D-galactose-induced aging. *Biol Pharm Bull.* (2025) 48:523–36. 10.1248/bpb.b24-00524 40335326

[98] Feng S Zou L Wang H He R Liu K Zhu H. RhoA/ROCK-2 pathway inhibition and tight junction protein upregulation by catalpol suppresses lipopolysaccaride-induced disruption of blood-brain barrier permeability. *Molecules.* (2018) 23:2371. 10.3390/molecules23092371 30227623 PMC6225311

[99] Hu S Wu Y Zhao B Hu H Zhu B Sun Z Panax notoginseng saponins protect cerebral microvascular endothelial cells against oxygen-glucose deprivation/reperfusion-induced barrier dysfunction via activation of PI3K/Akt/Nrf2 antioxidant signaling pathway. *Molecules.* (2018) 23:2781. 10.3390/molecules23112781 30373188 PMC6278530

[100] Chang H Tai Y Cherng Y Lin J Liu S Chen T Resveratrol attenuates high-fat diet-induced disruption of the blood-brain barrier and protects brain neurons from apoptotic insults. *J Agric Food Chem.* (2014) 62:3466–75. 10.1021/jf403286w 24694235

[101] Song C Gao C Wang Z. Grape-Seed-derived procyanidin attenuates chemotherapy-induced cognitive impairment by suppressing MMP-9 activity and related blood-brain-barrier damage. *Brain Sci.* (2022) 12:571. 10.3390/brainsci12050571 35624958 PMC9139059

[102] Dang Q Wu D Li Y Fang L Liu C Wang X Walnut-derived peptides ameliorate d-galactose-induced memory impairments in a mouse model via inhibition of MMP-9-mediated blood-brain barrier disruption. *Food Res Int.* (2022) 162:112029. 10.1016/j.foodres.2022.112029 36461249

[103] Fang W Sha L Kodithuwakku N Wei J Zhang R Han D Attenuated blood-brain barrier dysfunction by XQ-1H following ischemic stroke in hyperlipidemic rats. *Mol Neurobiol.* (2015) 52:162–75. 10.1007/s12035-014-8851-1 25128027

[104] Zhu J Jin Z Yang L Zhao C Hu J Chen J Ginkgolide B targets and inhibits creatine kinase B to regulate the CCT/TRiC-SK1 axis and exerts pro-angiogenic activity in middle cerebral artery occlusion mice. *Pharmacol Res.* (2022) 180:106240. 10.1016/j.phrs.2022.106240 35513225

[105] Choi M Lim C Lee B Cho S. Amelioration of brain damage after treatment with the methanolic extract of glycyrrhizae radix et rhizoma in mice. *Pharmaceutics.* (2022) 14:2776. 10.3390/pharmaceutics14122776 36559268 PMC9781260

[106] Zhao P Zhou R Zhu X Liu G Zhao Y Ma P Neuroprotective effects of lycium barbarum polysaccharide on focal cerebral ischemic injury in mice. *Neurochem Res.* (2017) 42:2798–813. 10.1007/s11064-017-2293-x 28508173

[107] Liu H Zang C Shang J Zhang Z Wang L Yang H *Gardenia jasminoides* J. Ellis extract GJ-4 attenuates hyperlipidemic vascular dementia in rats via regulating PPAR-γ-mediated microglial polarization. *Food Nutr Res.* (2022) 66:8101. 10.29219/fnr.v66.8101 35950104 PMC9338452

[108] Voirin A Perek N Roche F. Inflammatory stress induced by a combination of cytokines (IL-6, IL-17, TNF-α) leads to a loss of integrity on bEnd.3 endothelial cells in vitro BBB model. *Brain Res.* (2020) 1730:146647. 10.1016/j.brainres.2020.146647 31911168

[109] Anderson A Waithe O Seplovich G Olagunju O Greene C Singh A Regulation of BzATP-induced blood-brain barrier endothelial cell hyperpermeability by NLRP3 inflammasome inhibition. *Microcirculation.* (2025) 32:e70006. 10.1111/micc.70006 40052959 PMC11905927

[110] Zhou F Wang L Liu P Hu W Zhu X Shen H Puerarin protects brain tissue against cerebral ischemia/reperfusion injury by inhibiting the inflammatory response. *Neural Regen Res.* (2014) 9:2074–80. 10.4103/1673-5374.147934 25657724 PMC4316472

[111] Li B Zhang B Li Z Li S Li J Wang A Ginkgolide C attenuates cerebral ischemia/reperfusion-induced inflammatory impairments by suppressing CD40/NF-κB pathway. *J Ethnopharmacol.* (2023) 312:116537. 10.1016/j.jep.2023.116537 37094696

[112] Zhang S Chen Q Jin M Ren J Sun X Zhang Z Notoginsenoside R1 alleviates cerebral ischemia/reperfusion injury by inhibiting the TLR4/MyD88/NF-κB signaling pathway through microbiota-gut-brain axis. *Phytomedicine.* (2024) 128:155530. 10.1016/j.phymed.2024.155530 38493723

[113] Qin X Hua J Lin S Zheng H Wang J Li W Astragalus polysaccharide alleviates cognitive impairment and β-amyloid accumulation in APP/PS1 mice via Nrf2 pathway. *Biochem Biophys Res Commun.* (2020) 531:431–7. 10.1016/j.bbrc.2020.07.122 32800555

[114] Xiao L Wen H Peng S Chen B Tang B Liu B. Polygonatum polysaccharide ameliorates D-galactose-induced cognitive dysfunction in aging rats by inhibiting ferroptosis through activation of Nrf2. *Neurosci Lett.* (2024) 836:137873. 10.1016/j.neulet.2024.137873 38871020

[115] Ko G Kim J Jeon Y Lee D Baek H Chang K. Salvia miltiorrhiza alleviates memory deficit induced by ischemic brain injury in a transient MCAO mouse model by inhibiting ferroptosis. *Antioxidants.* (2023) 12:785. 10.3390/antiox12040785 37107160 PMC10135292

[116] Takizawa S Fukuyama N Hirabayashi H Kohara S Kazahari S Shinohara Y Quercetin, a natural flavonoid, attenuates vacuolar formation in the optic tract in rat chronic cerebral hypoperfusion model. *Brain Res.* (2003) 980:156–60. 10.1016/s0006-8993(03)03009-9 12865172

[117] Zhang J Zhang Y Liu Y Niu X. Naringenin attenuates cognitive impairment in a rat model of vascular dementia by inhibiting hippocampal oxidative stress and inflammatory response and promoting N-Methyl-D-aspartate receptor signaling pathway. *Neurochem Res.* (2022) 47:3402–13. 10.1007/s11064-022-03696-9 36028734

[118] Zhang G Zhao Z Gao L Deng J Wang B Xu D Gypenoside attenuates white matter lesions induced by chronic cerebral hypoperfusion in rats. *Pharmacol Biochem Behav.* (2011) 99:42–51. 10.1016/j.pbb.2011.03.019 21459105

[119] Zhou X Wang H Wu B Cheng C Xiao W Wang Z Ginkgolide K attenuates neuronal injury after ischemic stroke by inhibiting mitochondrial fission and GSK-3β-dependent increases in mitochondrial membrane permeability. *Oncotarget.* (2017) 8:44682–93. 10.18632/oncotarget.17967 28591721 PMC5546510

[120] Su S Cheng C Tsai T Hsieh C. Paeonol protects memory after ischemic stroke via inhibiting β-secretase and apoptosis. *Evid Based Complement Alternat Med.* (2012) 2012:932823. 10.1155/2012/932823 22474531 PMC3312264

[121] Le X Nguyet Pham H Van Nguyen T Minh Nguyen K Tanaka K Fujiwara H Protective effects of *Bacopa monnieri* on ischemia-induced cognitive deficits in mice: the possible contribution of bacopaside I and underlying mechanism. *J Ethnopharmacol.* (2015) 164:37–45. 10.1016/j.jep.2015.01.041 25660331

[122] He Y Chen S Tsoi B Qi S Gu B Wang Z Alpinia oxyphylla Miq. and its active compound P-coumaric acid promote brain-derived neurotrophic factor signaling for inducing hippocampal neurogenesis and improving post-cerebral ischemic spatial cognitive functions. *Front Cell Dev Biol.* (2020) 8:577790. 10.3389/fcell.2020.577790 33537297 PMC7849625

[123] Ju D Kuo W-W Ho T-J Chang R-L Lin W-T Day CH Bioactive peptide VHVV upregulates the long-term memory-related biomarkers in adult spontaneously hypertensive rats. *Int J Mol Sci.* (2019) 20:3069. 10.3390/ijms20123069 31234585 PMC6627188

[124] Wang X Li J Qian L Zang X Zhang S Wang X Icariin promotes histone acetylation and attenuates post-stroke cognitive impairment in the central cholinergic circuits of mice. *Neuroscience.* (2013) 236:281–8. 10.1016/j.neuroscience.2012.12.074 23370322

[125] Li Y Wu H Liu M Zhang Z Ji Y Xu L Polysaccharide from Polygala tenuifolia alleviates cognitive decline in Alzheimer’s disease mice by alleviating Aβ damage and targeting the ERK pathway. *J Ethnopharmacol.* (2024) 321:117564. 10.1016/j.jep.2023.117564 38081400

[126] Chun L Ramachandran R Othman S Has A George A Mat N *Persicaria minor* ameliorates the cognitive function of chronic cerebral hypoperfusion rats: metabolomic analysis and potential mechanisms. *Behav Brain Res.* (2023) 447:114423. 10.1016/j.bbr.2023.114423 37030545

[127] Igarashi K Kurata D. Effect of high-oleic peanut intake on aging and its hippocampal markers in senescence-accelerated mice (SAMP8). *Nutrients.* (2020) 12:3461. 10.3390/nu12113461 33187266 PMC7697529

[128] Xu X Zheng X Zhou Q Li H. Inhibition of excitatory amino acid efflux contributes to protective effects of puerarin against cerebral ischemia in rats. *Biomed Environ Sci.* (2007) 20:336–42. 17948770

[129] Rishitha N Muthuraman A. Preventative effects of alpha-naphtho flavone in vascular dementia. *Front Biosci.* (2020) 12:79–94. 10.2741/E858 31585870

[130] Sherwin E Sandhu K Dinan T Cryan J. May the force be with you: the light and dark sides of the microbiota-gut-brain axis in neuropsychiatry. *CNS Drugs.* (2016) 30:1019–41. 10.1007/s40263-016-0370-3 27417321 PMC5078156

[131] Li G Cheong K He Y Liew A Huang J Huang C Hylocereus polyrhizus pulp residues polysaccharide alleviates high-fat diet-induced obesity by modulating intestinal mucus secretion and glycosylation. *Foods.* (2025) 14:2708. 10.3390/foods14152708 40807645 PMC12345779

[132] Yang S Wang L Liang X Pei T Zeng Y Xie B Radix hedysari polysaccharides modulate the gut-brain axis and improve cognitive impairment in SAMP8 mice. *Int J Biol Macromol.* (2025) 306:141715. 10.1016/j.ijbiomac.2025.141715 40044002

[133] Song J Li M Kang N Jin W Xiao Y Li Z Baicalein ameliorates cognitive impairment of vascular dementia rats via suppressing neuroinflammation and regulating intestinal microbiota. *Brain Res Bull.* (2024) 208:110888. 10.1016/j.brainresbull.2024.110888 38295883

[134] Zheng L Meng L Liang H Yang J. Sanhua decoction: current understanding of a traditional herbal recipe for stroke. *Front Neurosci.* (2023) 17:1149833. 10.3389/fnins.2023.1149833 37123364 PMC10133510

[135] Thongwong P Wattanathorn J Thukham-mee W. A novel supplement consisting of rice, silkworm pupae and a mixture of ginger and holy basil improves post-stroke cognitive impairment. *Nutrients.* (2024) 16:4144. 10.3390/nu16234144 39683536 PMC11644478

[136] Tang C Wang C Wang J Wang Q Li S Wang H Short-chain fatty acids ameliorate depressive-like behaviors of high fructose-fed mice by rescuing hippocampal neurogenesis decline and blood-brain barrier damage. *Nutrients.* (2022) 14:1882. 10.3390/nu14091882 35565849 PMC9105414

[137] Wei F Zhou J Pan L Shen M Niu D Zeng Z Integrative microbiomics, proteomics and lipidomics studies unraveled the preventive mechanism of Shouhui Tongbian Capsules on cerebral ischemic stroke injury. *J Ethnopharmacol.* (2025) 337:118874. 10.1016/j.jep.2024.118874 39362332

[138] Zhai L Zheng Y Lo C Xu S Jiang X Liu Q Butyrate-producing commensal bacteria mediates the efficacy of herbal medicine JCM-16021 on abdominal pain in diarrhea-predominant irritable bowel syndrome: a randomized clinical trial. *Phytomedicine.* (2025) 145:157040. 10.1016/j.phymed.2025.157040 40639240

[139] Elberry D Gamal M Gawish Z Hegazy E Hosny S Rashed L Amelioration of gut dysbiosis-induced cognitive deterioration by repeated administration of human clostridium butyricum: targeting intestinal and blood–brain barrier. *Future J Pharmaceutical Sci.* (2025) 11:87. 10.1186/s43094-025-00836-0

[140] Li Y Xu Z Du P Gao J Wang S Pang X Methodological challenges in pilot trials of herbal medicine: barriers to evidence-based practice. *J Clin Epidemiol.* (2025) 182:111754. 10.1016/j.jclinepi.2025.111754 40081675

[141] Kim H Kim S Gil W Yang C. Exploring the therapeutic potential of phytochemicals: challenges and strategies for clinical translation. *Phytomedicine.* (2025) 145:157090. 10.1016/j.phymed.2025.157090 40716124

[142] Chen G Lu Y Yang M Li J Fan B. Medicinal uses, pharmacology, and phytochemistry of Convolvulaceae plants with central nervous system efficacies: a systematic review. *Phytother Res.* (2018) 32:823–64. 10.1002/ptr.6031 29356185

[143] Li Y Yao Y Cao X Yi N Chen A Li J Clinical efficacy of Danshen preparation in the treatment of vascular cognitive impairment: a systematic review and meta-analysis. *Front Aging Neurosci.* (2022) 14:1090665. 10.3389/fnagi.2022.1090665 36742208 PMC9895948

[144] Swartz R Longman R Lindsay M Lund R Ganesh A Eskes G Canadian stroke best practice recommendations: vascular cognitive impairment, 7th edition practice guidelines update, 2024. *Alzheimers Dement.* (2025) 21:e14324. 10.1002/alz.14324 39822128 PMC11772713

[145] Indrayanto G. Regulation and standardization of herbal drugs: current status, limitation, challenge’s and future prospective. *Profiles Drug Subst Excip Relat Methodol.* (2024) 49:153–99. 10.1016/bs.podrm.2023.11.003 38423707

[146] Zhang C Xue M Zhang H Li J He M. Functional brain changes in vascular cognitive impairment: a whole brain ALE meta-analysis. *Front Aging Neurosci.* (2025) 17:1521457. 10.3389/fnagi.2025.1521457 40538419 PMC12176746

[147] Mok V Cai Y Markus H. Vascular cognitive impairment and dementia: mechanisms, treatment, and future directions. *Int J Stroke.* (2024) 19:838–56. 10.1177/17474930241279888 39283037 PMC11490097

[148] Candelario-Jalil E Thompson J Taheri S Grossetete M Adair J Edmonds E Matrix metalloproteinases are associated with increased blood-brain barrier opening in vascular cognitive impairment. *Stroke.* (2011) 42:1345–50. 10.1161/STROKEAHA.110.600825 21454822 PMC3119779

[149] Lin L Huang Y Xu J Han J Wu S Jin Y Mechanistic insights and translational therapeutics of neurovascular unit dysregulation in vascular cognitive impairment. *J Integr Neurosci.* (2025) 24:40091. 10.31083/JIN40091 40919631

[150] Bayraktar D Ertaş B Aydın Y Şener G. Carvacrol improves cognitive dysfunction by decreasing amyloid-β accumulation and regulating neuroinflammation in ovariectomized renovascular hypertensive rats. *Naunyn Schmiedebergs Arch Pharmacol.* (2025) 398:2797–813. 10.1007/s00210-024-03442-8 39283526

[151] Chen X Luo X Chen L Liu H Yin X Chen Z. Developments in MRI radiomics research for vascular cognitive impairment. *Insights Imaging.* (2025) 16:146. 10.1186/s13244-025-02026-1 40593438 PMC12214101

[152] Shadrack S Wang Y Mi S Lu R Zhu Y Tang Z Enhancing bioavailability and functionality of plant peptides and proteins: a review of novel strategies for food and pharmaceutical applications. *Food Chem.* (2025) 485:144440. 10.1016/j.foodchem.2025.144440 40288337

[153] Shen D Kunze K Thummel K. Enzyme-catalyzed processes of first-pass hepatic and intestinal drug extraction. *Adv Drug Deliv Rev.* (1997) 27:99–127. 10.1016/s0169-409x(97)00039-2 10837554

[154] Zhang L Pan Siu A Lin G Zuo Z. Intestinal absorbability of three Radix Puerariae isoflavones including daidzein, daidzin and puerarin. *Chin Med.* (2011) 6:41. 10.1186/1749-8546-6-41 22108408 PMC3253046

[155] Rosiak N Tykarska E Miklaszewski A Pietrzak R Cielecka-Piontek J. Enhancing the solubility and dissolution of apigenin: solid dispersions approach. *Int J Mol Sci.* (2025) 26:566. 10.3390/ijms26020566 39859284 PMC11766082

[156] Nguyen T Duong V Maeng H. Pharmaceutical formulations with P-Glycoprotein inhibitory effect as promising approaches for enhancing oral drug absorption and bioavailability. *Pharmaceutics.* (2021) 13:1103. 10.3390/pharmaceutics13071103 34371794 PMC8309061

[157] Ebinger M Uhr M. ABC drug transporter at the blood-brain barrier: effects on drug metabolism and drug response. *Eur Arch Psychiatry Clin Neurosci.* (2006) 256:294–8. 10.1007/s00406-006-0664-4 16783492

[158] Boulton D Walle U Walle T. Extensive binding of the bioflavonoid quercetin to human plasma proteins. *J Pharm Pharmacol.* (1998) 50:243–9. 10.1111/j.2042-7158.1998.tb06183.x 9530994

[159] Yang M Wei X Pan K Zhou Z Liu Y Lv X Brain-targeted ginkgolide B-modified carbonized polymer dots for alleviating cerebral ischemia reperfusion injury. *Biomater Sci.* (2023) 11:3998–4008. 10.1039/d2bm02013k 37128751

[160] Li Y Zhang M Li S Zhang L Kim J Qiu Q Selective ischemic-hemisphere targeting Ginkgolide B liposomes with improved solubility and therapeutic efficacy for cerebral ischemia-reperfusion injury. *Asian J Pharm Sci.* (2023) 18:100783. 10.1016/j.ajps.2023.100783 36891470 PMC9986716

[161] Yang L Wang Y Zheng G Li Z Mei J. Resveratrol-loaded selenium/chitosan nano-flowers alleviate glucolipid metabolism disorder-associated cognitive impairment in Alzheimer’s disease. *Int J Biol Macromol.* (2023) 239:124316. 10.1016/j.ijbiomac.2023.124316 37004937

[162] Su Y Guo C Chen Q Guo H Wang J Mu K Construction of bionanoparticles based on Angelica polysaccharides for the treatment of stroke. *Nanomedicine.* (2022) 44:102570. 10.1016/j.nano.2022.102570 35623564

[163] Li Z Zheng G Wang N Liang H Li C Wang Y Flower-like brain targeted selenium nanocluster lowers the chlorogenic acid dose for ameliorating cognitive impairment in APP/PS1 mice. *J Agric Food Chem.* (2023) 71:2883–97. 10.1021/acs.jafc.2c06809 36722770

[164] Liu Y Rao L Zhang H Cen Y Cheng K. Conjugation of vitamin E-TPGS and guar gum to carry borneol for enhancing blood-brain barrier permeability. *J Biomater Appl.* (2018) 33:590–8. 10.1177/0885328218799551 30208770

[165] Huang L Liu Y Li L Zhang M Miao X Liang Z *Momordica charantia* small extracellular vesicles mitigate neuronal ferroptosis by inhibition of GPX4 ubiquitination in ischemic stroke. *Phytomedicine.* (2025) 148:157298. 10.1016/j.phymed.2025.157298 41033098

[166] Zhang S Liang Z Wu C Qin Z Wei X Liu Y Houttuynia cordata Thunb-derived extracellular vesicle-like particles alleviate ischemic brain injury by miR159a targeting ACSL4 to suppress ferroptosis. *Chin Med.* (2025) 20:141. 10.1186/s13020-025-01193-z 40890801 PMC12400549

[167] Xie X Zhou X Chen W Deng X Jiang J Wen Z Hybrid exosome-liposome nanoparticles for dual modulation of neuroinflammation and lipid metabolism in ischemic stroke. *ACS Nano.* (2025) 19:33567–86. 10.1021/acsnano.5c11417 40948043

[168] Oller-Salvia B Sánchez-Navarro M Giralt E Teixidó M. Blood-brain barrier shuttle peptides: an emerging paradigm for brain delivery. *Chem Soc Rev.* (2016) 45:4690–707. 10.1039/c6cs00076b 27188322

[169] Chen D Jiang H Sun L Nurzat Y Qin H Zhao Z Neuron-targeted ROS-responsive liposomes for puerarin delivery remodel ischemic microenvironment via microglial modulation and neurovascular regeneration. *J Nanobiotechnology.* (2025) 23:677. 10.1186/s12951-025-03730-2 41084040 PMC12519847

[170] Yang Q Li R Hong Y Liu H Jian C Zhao S. Curcumin-loaded gelatin nanoparticles cross the blood-brain barrier to treat ischemic stroke by attenuating oxidative stress and neuroinflammation. *Int J Nanomedicine.* (2024) 19:11633–49. 10.2147/IJN.S487628 39553455 PMC11568047

[171] Raghavan S Graff-Radford J Hofrenning E Fought A Reid R Kamykowski M Plasma NfL and GFAP for predicting VCI and related brain changes in community and clinical cohorts. *Alzheimers Dement.* (2025) 21:e70381. 10.1002/alz.70381 40566826 PMC12198477

[172] Edwards N Lao P Alshikho M Mazen J Huber B Hale C Alzheimer disease, vascular disease, and blood-brain barrier permeability biomarkers in middle-aged adults. *Neurology.* (2025) 105:e214220. 10.1212/WNL.0000000000214220 41052375 PMC12591055

[173] Halloway S Aggarwal N Arfanakis K Sacks F Barnes L Dhana K. Effect modifiers of the MIND diet for cognition in older adults: the MIND diet trial. *Alzheimers Dement.* (2025) 21:e70731. 10.1002/alz.70731 41058007 PMC12504047

